# Contrasted Patterns of Crossover and Non-crossover at *Arabidopsis thaliana* Meiotic Recombination Hotspots

**DOI:** 10.1371/journal.pgen.1003922

**Published:** 2013-11-14

**Authors:** Jan Drouaud, Hossein Khademian, Laurène Giraut, Vanessa Zanni, Sarah Bellalou, Ian R. Henderson, Matthieu Falque, Christine Mézard

**Affiliations:** 1 Institut Jean-Pierre Bourgin UMR1318 INRA-AgroParisTech, Versailles, France; 2 Department of Plant Sciences, University of Cambridge, Cambridge, United Kingdom; 3 UMR de Génétique Végétale du Moulon, INRA/CNRS/Univ Paris-Sud/AgroParisTech, Ferme du Moulon, Gif sur Yvette, France; The Pennsylvania State University, United States of America

## Abstract

The vast majority of meiotic recombination events (crossovers (COs) and non-crossovers (NCOs)) cluster in narrow hotspots surrounded by large regions devoid of recombinational activity. Here, using a new molecular approach in plants, called “pollen-typing”, we detected and characterized hundreds of CO and NCO molecules in two different hotspot regions in *Arabidopsis thaliana*. This analysis revealed that COs are concentrated in regions of a few kilobases where their rates reach up to 50 times the genome average. The hotspots themselves tend to cluster in regions less than 8 kilobases in size with overlapping CO distribution. Non-crossover (NCO) events also occurred in the two hotspots but at very different levels (local CO/NCO ratios of 1/1 and 30/1) and their track lengths were quite small (a few hundred base pairs). We also showed that the ZMM protein MSH4 plays a role in CO formation and somewhat unexpectedly we also found that it is involved in the generation of NCOs but with a different level of effect. Finally, factors acting in *cis* and in *trans* appear to shape the rate and distribution of COs at meiotic recombination hotspots.

## Author Summary

During meiosis, genomes are reshuffled by recombination between homologous chromosomes. Reciprocal recombination events called crossovers are clustered in several kilobase-wide regions called hotspots, where their frequency is greatly enhanced compared to adjacent regions. Our understanding of hotspot organization is based on analyses performed in only a few species and rules differ between species. For the first time, hundreds of recombination events were analyzed in *Arabidopsis thaliana* revealing several new features: (i) crossovers are concentrated in hotspots where their rate reaches up to 50 times the genome average; (ii) non-crossovers events, (also called gene conversions not associated with crossovers) also occur in hotspots but at very different levels; and (iii) in the absence of the recombination protein MSH4, the crossover rate is dramatically reduced (70 times less than the wild-type level) and the crossover distribution within a hotspot is also largely modified; unexpectedly, the non-crossover rate was also altered (15% of the wild-type level at a hotspot). Finally we showed that factors acting in *cis* and in *trans* may influence the level and distribution of crossovers at and between hotspots.

## Introduction

Meiosis reduces the level of ploidy by half. To fulfill this goal, homologous chromosomes (homologs) are segregated at the first meiotic division. In most eukaryotes, accurate segregation is ensured by the formation of at least one reciprocal recombination event or crossover (CO) between the chromatids of homologs [1]. In addition to this crucial mechanical role, COs increase genetic diversity by reshuffling alleles along the genome.

In all eukaryotes, CO distribution along chromosomes is not homogeneous. COs tend to be clustered in narrow regions (two to three kilobases wide) called hotspots where CO frequencies are greatly enhanced compared to large adjacent regions almost devoid of any recombinational activity [2]. For example, 80% of all recombination occurs in 10 to 20% of the human genome [3].

**Figure 1:**
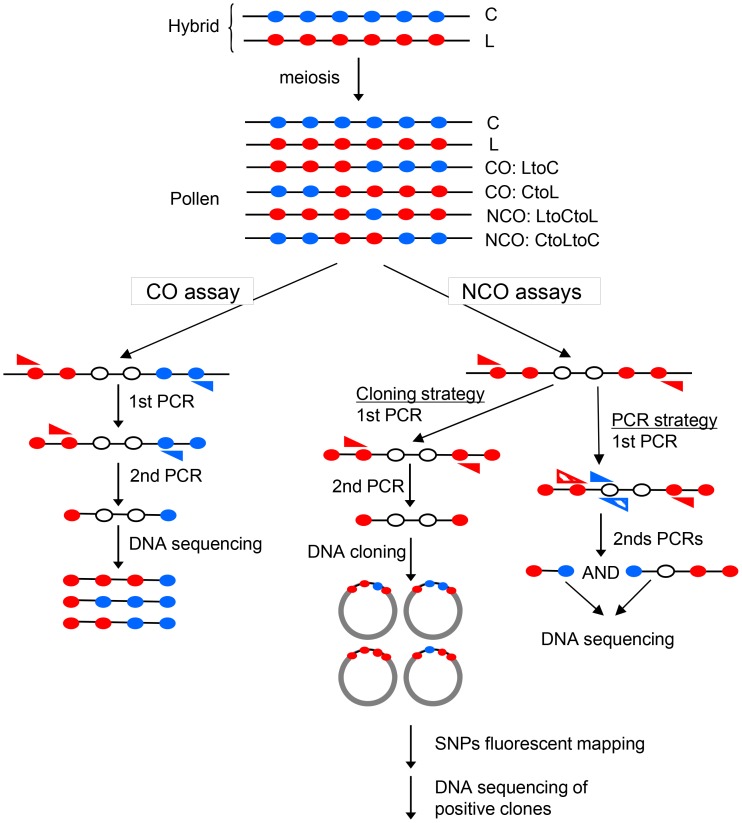
Specific detection of CO and NCO molecules in genomic DNA extracted from pollen. The F1 hybrid contains one allele from each parent at each locus. Filled circles represent polymorphisms on the C (Col, blue) or L (Ler, red) chromosomes. After meiosis, in DNA extracted from pollen, there are either non-recombinant molecules (C, L) or CO (‘LtoC’ or ‘CtoL’) or NCOs (‘LtoCtoL’ or ‘CtoLtoC’) in various proportions depending on the locus studied. To detect CO molecules, two rounds of allele-specific PCR were carried out with allele-specific oligonucleotides (ASOs, blue and red triangles) on pools of genomic DNA. To detect NCO molecules, alleles containing polymorphisms from one specific parent on each side were first amplified by one or two rounds of allele-specific PCR. Then the region of interest was cloned in *E.coli* and interesting SNPs were genotyped using fluorescent mapping as described [31]. Alternatively, two rounds of complementary allele-specific PCR were performed to analyze the status of interesting SNPs. A NCO event was scored when both PCRs were positive.

The molecular organization of hotspots has been deciphered in the two yeasts *Saccharomyces cerevisiae* and *Schizosacchamomyces pombe*. As most of the proteins involved in the meiotic recombination process are evolutionary conserved, it is thought that their basic features are similar in all eukaryotes: meiotic recombination is initiated by DNA double-strand breaks (DSBs) formed early in meiotic prophase at the leptotene stage by the Spo11 protein [4], [5]. The initiating DSBs are repaired preferentially by interactions with a non-sister chromatid. After completion of the DSB repair process, both COs and non-reciprocal recombination events, also called non-crossovers (NCOs) can be recovered [6] (Figure 1). COs and NCOs cluster around the DSBs sites in hotspot regions. CO rates peak at the center of the hotspots and then decrease on either side of this region [2], [7].

Most studies of meiotic recombination hotspots characterized COs whereas because NCOs are difficult to detect these have rarely been analyzed. COs *per se* are the major determinant of linkage disequilibrium (non-random association of genetic markers) breakdown. In addition, the gene conversion tracks contained in both COs and NCOs shape the haplotype landscape. Indeed, CO associated gene conversion events soften the boundaries between haplotype blocks while NCOs create holes within blocks [8]. Thus it is important to appreciate both phenomena as they have implications for genetic association analyses. The ratio of COs to NCOs varies from one hotspot to another, from 14∶0 to 0∶7 in *S. cerevisiae* with a very low CO to NCO ratio next to telomeres (excess of NCOs) and repression of both CO and NCOs close to centromeres [9]. The CO to NCO ratio is also extremely variable in human and mice from more than 12∶1 to 1∶10 [10]. In plants, NCOs have been detected at only a few loci and mainly in maize: at the bronze loci, the CO to NCO ratio varies from 30∶1 to 1∶6 depending on the presence or not of large indels in the region [11]. In *Arabidopsis*, to date little information is available. Using antibodies directed against the meiotic DSB repair protein DMC1, DSB sites have been estimated to be between 100 to 200 per meiosis in male meiosis of various accessions [12]–[14] whereas COs vary between 7 to 11 depending on the accessions studied [15]. If these breaks are mainly repaired as NCOs, there should be a large excess (at least 20 times) of NCO events compared to COs but the relative ratio of DSBs repaired on homologous versus sister chromosomes is totally unknown in *Arabidopsis* as in other higher eukaryotes. Several recent studies have tried to tackle the question of the meiotic NCOs rate in *A. thaliana*. Genome-wide studies using Next Generation Sequencing (NGS) gave contradictory results with NCOs found to be either a rare meiotic event [16] or, in striking contrast, several hundred times more frequent than COs [17]. Another study analyzed gene conversion rates at several loci but found only one NCO event at one loci among more than 10^6^ tetrads, the others being associated with COs [18]. Thus, NCO features are poorly understood in plants.

Meiotic hotspots are under the control of a series of genes that channel DSB repair toward different pathways. In most organisms, two CO pathways coexist. One is dependent on a group of proteins called ZMM [19]. When one of these proteins is absent, there is a dramatic reduction in COs and the remaining COs do not exhibit interference (a phenomena described by H. J. Muller in 1916 [20], where, on the same molecule, multiple COs are more widely spaced than expected if they were placed randomly [21]). A second pathway is controlled, at least partially, by the Mus81 complex and COs in this pathway are interference-free. The ratio of interfering to non-interfering COs varies considerably from one species to another. *Caenorabditis elegans* has only interfering COs while all COs in *S. pombe* are interference-free. Both pathways appear to contribute equally in *S. cerevisiae*. However, in most higher eukaryotes, it seems that a vast majority of COs belong to the interfering pathway: 90 to 95% in mice and Humans, 85% in *A. thaliana* [21]. Up to now, none of the ZMM proteins have been shown to play a role in the formation or processing of NCOs.

Our current understanding of the organization of hotspots benefits from analyses in a few species, essentially fungi and mammals. The findings of these studies suggest that there are similarities but also differences in the formation and control of these hotspots. We characterized hotspot regions in a very different model, the plant *A. thaliana*. We set up a “pollen-typing” molecular approach (see Results), based on the “sperm typing” technique developed previously in mammals [22], that allowed us to detect and characterize hundreds of CO and NCO molecules at different hotspots. We obtained evidence for the existence of factors acting in *cis* and in *trans* that appear to influence CO rate and distribution and our data also suggest a role for the ZMM protein MSH4 in NCO formation.

## Results

### Detection of COs at three meiotic hotspots by “pollen-typing”

**Figure 2:**
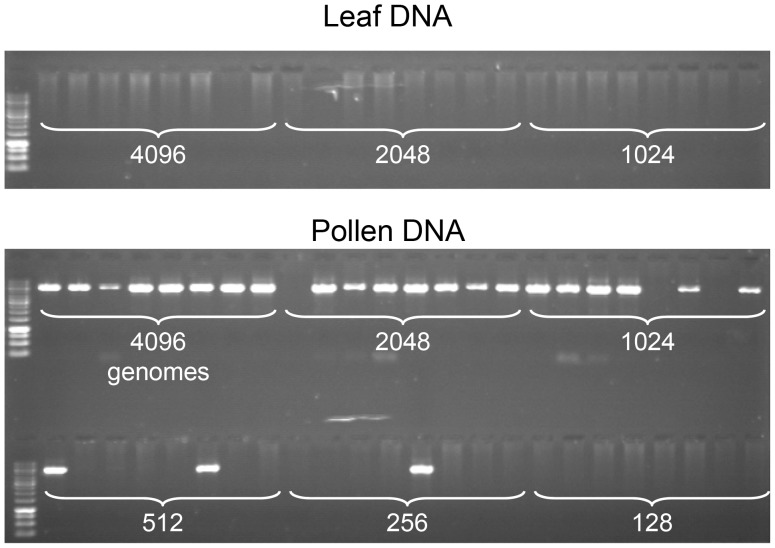
Specific detection of CO molecules in genomic DNA extracted from pollen. PCR was performed with allele-specific oligonucleotides (ASOs) designed for the amplification of CO molecules (see Material and Methods, Figure 1), using decreasing amounts of genomic DNA (the number of template molecules is indicated on the photograph) extracted from F1 Col x L*er* hybrid plants, either from leaf (two top rows) or pollen (two bottom rows). Eight aliquot reactions were carried out for each dilution. No PCR products were amplified from leaf. However, using equivalent low concentrations of DNA extracted from pollen (4096 molecules and less), CO molecules were strongly and specifically amplified .

A previous study of CO distribution over the entire *A. thaliana* chromosome 4, in large populations of hybrids between “Columbia-0” (Col) and “Landsberg *erecta*-4” (L*er*), identified the regions 14a and 130× as good candidates for true CO hotspots [23]. In these two regions of a few kilobases only, CO rates were found to be 20 to 30 times higher than the chromosomal average (4.8 cM/Mb). However, classical genetic techniques could not be used to further investigate these regions, as several tens of thousands of plants would have been needed to obtain enough COs to characterize these regions. Thus we set up a “pollen-typing” technique (see Materials and Methods; Figure 1; [24]), which parallels the “sperm-typing” technique used for hotspot studies in mice and humans [25]–[27]. Briefly (see Material and Methods for more details), genomic DNA (gDNA) was extracted from millions of pollen grains collected from a series of F1 ColxL*er* hybrids and precisely quantified by PCR (see Material and Methods). Taking into account the CO frequency estimated by our genetic map, the gDNA was then diluted to obtain less than one putative recombinant molecule per PCR reaction. Recombinant products were detected by two rounds of allelic PCRs (Figure 1). When an appropriate dilution was reached, a large series of PCR reactions was performed to detect the presence or absence of at least one template molecule in each reaction. CO rates could then be estimated using the Bayesian inference approach described in the Material and Methods. The meiotic origin of these molecules was assessed with control reactions carried out in parallel with pollen and leaf (somatic) DNA. Using the same amounts of gDNA, CO molecules could be detected in pollen DNA, but never in leaf DNA (Figure 2). Indeed, when the few positive PCR products amplified from a large input of leaf genomes (at least 15 times more genomes than in the PCR reaction used to detect recombinant molecules on pollen DNA) were sequenced, they were found to result from non-specific amplification. They did not correspond to a single locus of the *Arabidopsis* genome but rather to a complex mixture of loci from different chromosomes (data not shown) in contrast to products obtained from pollen DNA (see below).

We were able to amplify and characterize hundreds of recombinant CO molecules at both regions in gDNA extracted from the pollen of ColxL*er* hybrid plants (167 and 104 COs at 130× and 14a respectively). The CO rate in pollen gDNA was 0.55% (Confidence Intervals (CI): 0.29–0.95) at 14a, and 0.53% (CI:0.34–0.78) at 130×. The recombinant molecules were confirmed by sequencing and their exchange point mapped precisely. All CO molecules characterized contained a single transition between the parental haplotypes.

**Figure 3:**
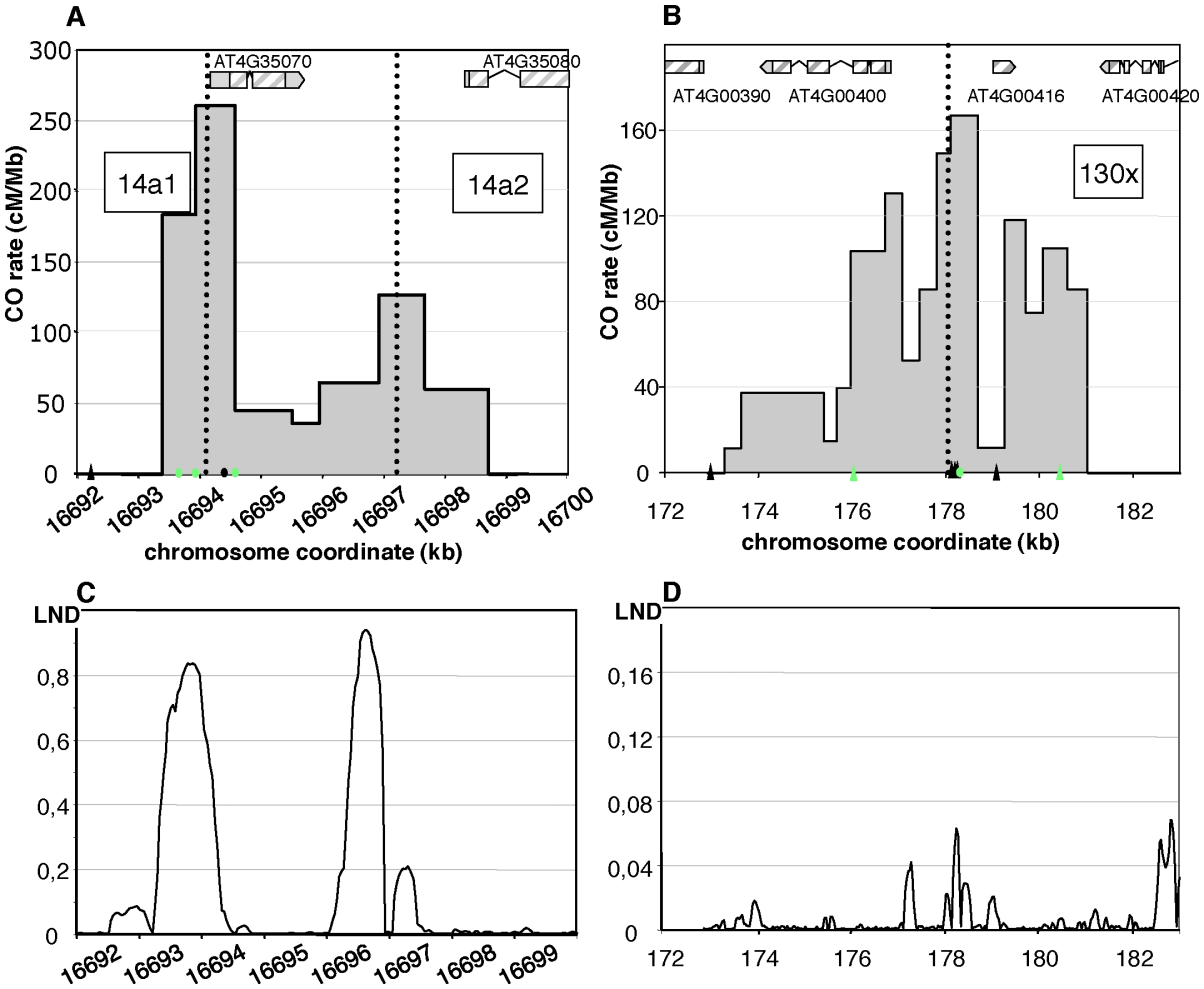
Distribution of CO rates across the 14a (A) and 130× (B) hotspots. One hundred and four COs were analyzed at 14a (62 at 14a1 and 42 at 14a2) and 167 at 130×. Grey dotted vertical lines: median positions of the hotspots. On the x axis: triangles are positions of insertions-deletions larger than 7 bp (from the left to the right of 130×: 20, 13, 12, 70, 10, 7 and, 11 bp); green triangles are positions of indels used to detect NCOs; green dots are SNPs used to detect NCOs; the black dot on the x axis in (A) is the position of the microsatellite. Gene structures along the hotspot regions are displayed on top of graphical areas (light hashed grey: exons, dark grey: UTR, broken lines: introns). (C)(D) Low nucleosome density (LND) at 14a and 130×. LND is from published nucleosomal DNA microarray hybridization experiments, where a high signal represents an absence of nucleosomal DNA [28].

Two distinct CO peaks were observed in the 14a region (subsequently referred to as 14a1 and 14a2), which both fit a Gaussian distribution (Figure 3A). The width of the hotspots within which 95% of COs occurred (determined by best fit normal distributions, see Material and Methods) was 1,475 bp and 3,775 bp for 14a1 and 14a2, respectively. Their respective medians are 3,047 bp apart, with a “valley” in between where just a few COs were detected. The CO frequency was null on either side of this region (Figure 3A). At 14a1 and 14a2, CO rates peak at 261 and 127 cM/Mb, respectively (54 and 26 times the chromosomal average). To investigate the relationship between CO frequency at 14a and chromatin we plotted published low nucleosome density (LND) data over the same region (Figure 3C), where a high signal represents an absence of nucleosomal DNA [28]. Regions of LND are typically observed at the 5′ of genes coincident with transcriptional start sites (TSS). Consistent with this observation, the LND peaks were located upstream of the two genes within 14a. Strikingly we observed an overlap of the 14a CO frequency peaks and the LND peaks (Figure 3C) which suggest that DNA accessibility promotes COs at the 14a hotspot.

At 130×, the CO rate was also null on both sides of the region and reached a maximum (close to the center) at 167 cM/Mb, which is 35 times the chromosomal average (Figure 3B). The distribution of COs differed from that observed in 14a: it was broad (more than 7 kb) and irregular with alternating “peaks” and “valleys” (Figure 3B) which does not fit well with a unique Gaussian curve. Interestingly, there was little correlation between CO peaks and LND at 130× (Figure 3D), suggesting that hotspots exist, which have different relationships to nucleosome density. Altogether, both the CO rate and distribution at 14a and 130× clearly indicate the existence of hotspots in *A. thaliana*.

**Figure 4:**
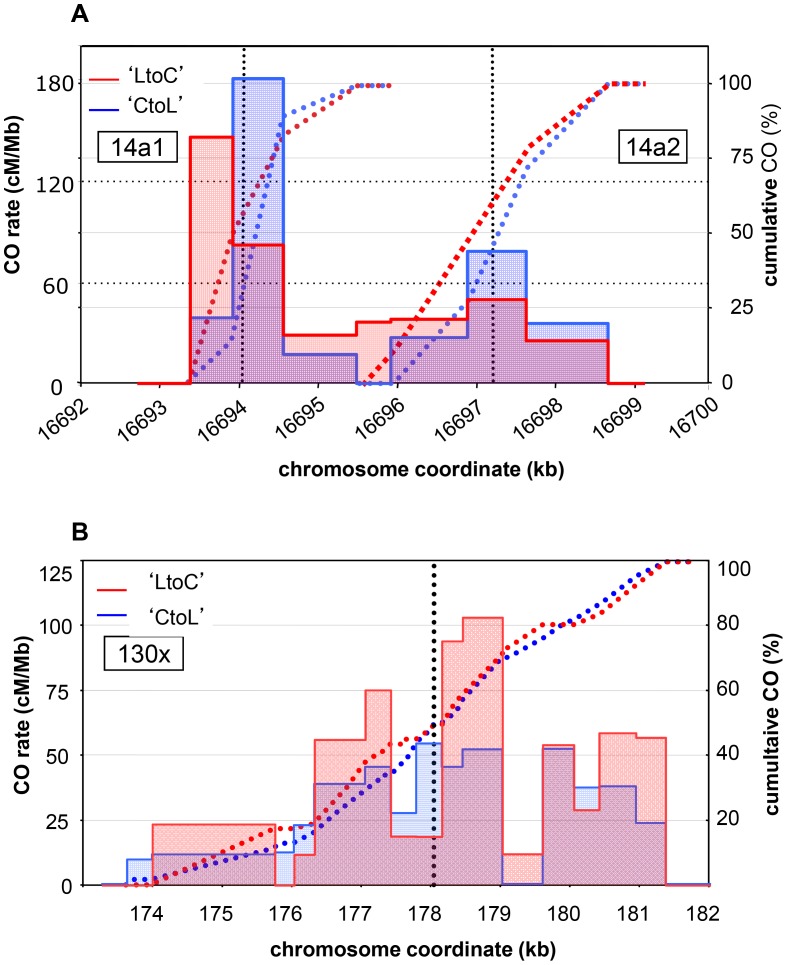
CO distribution and cumulative CO rates for reciprocal orientations at 14a (A) and 130× (B). Twenty nine ‘CtoL’ and 33 ‘LtoC’ COs were analyzed at 14a1, 23 and 19 at 14a2, and 75 and 92 at 130×. The ‘LtoC’ distribution is presented in red and the ‘CtoL’ in blue. For all three hotspots and both reciprocal orientations, cumulated (from left to right) relative CO rates were scored at successive polymorphic sites along the region. Blue curves: cumulated relative ‘CtoL’ CO rates. Red curves: cumulated relative ‘LtoC’ CO rates. Blue histogram: distribution of ‘CtoL’ CO rates. Red histogram: distribution of ‘LtoC’ CO rates. Grey dotted vertical lines: median positions of the hotspots.

We then looked at the distribution of exchange points in the recombinant molecules in each orientation at both loci (Figure 4). At the 14a hotspots (14a1+14a2), discrepancies between CO distribution in the reciprocal orientations “Col to L*er*” and “L*er* to Col” (i.e. ‘CtoL’ and ‘LtoC’) were observed: ‘LtoC’ exchanges were shifted to the left of ‘CtoL’ exchanges (Figure 4A). A comparison of cumulative CO distribution patterns showed that this leads to an excess of the Col allele at the center of both hotspots (Figure 4A). At 14a1, the Col allele was over-transmitted by 68% and this difference was highly significant (*p-value* = 0.00111), while it was only barely significant (*p-value* = 0.0734) for the 14a2 hotspot, probably due to the lower CO number. These patterns are consistent with the hypothesis that the L*er* allele has a stronger initiation activity than the Col allele at these hotspots [29]. The mean position of the two reciprocal distributions ‘CtoL’ and ‘LtoC’ was separated on average by 213 bp and 483 bp for the 14a1 and 14a2 hotspots respectively. In contrast, at the 130× hotspots both alleles appeared equally proficient at initiating recombination (Figure 4B).

### Detection of NCO events at meiotic recombination hotspots

At meiotic recombination hotspots, DSBs are repaired as either COs or NCOs. In plants, very few meiotic NCOs have been characterized because of the difficulty in detecting molecular events unless they are linked to a phenotypic change. We characterized NCO events at both the 14a1 and 130× hotspots, with different molecular approaches adapted to the polymorphisms available at each hotspot (Figure 1; see Material and Methods; [30]).

**Figure 5:**
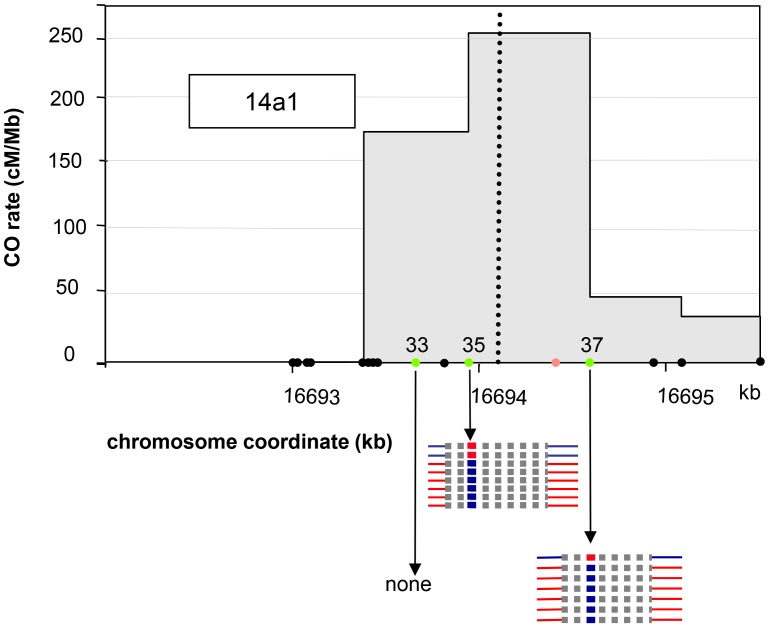
NCO events at 14a1. The SNPs #33, #35 and #37 are indicated by filled green circles. The polymorphisms are indicated by filled black circles along the chromosome coordinate axes. The position of the microsatellite located between #35 and #37 is indicated as an orange circle. Thick blue or red horizontal lines: converted SNPs, Thick dotted grey horizontal lines: interval in which NCO tract ends are located. Blue: Col; Red: L*er.*

For 14a1, the polymorphisms at the center of the hotspot were not suitable for a pollen typing strategy. Thus we used a cloning strategy based on a method described in [31] (Figure 1; see Material and Methods, [30]): after two rounds of allele-specific PCR performed on pollen DNA, the fragment corresponding to the 14a1 hotspot region was cloned. 3000 clones were individually genotyped at three SNPs: two (#35 and #37) located on opposite sides of the center of the hotspot and one (#33) on the left border (Figure 5). For the control reaction, a similar series of PCRs, cloning and genotyping was performed with DNA extracted from F1 ColxL*er* leaves. Positives clones obtained with pollen DNA were sequenced (see Material and Methods; Figure 1). Among 3,000 molecules tested, 8 and 7 NCO events were detected at polymorphism #35 and #37 respectively, and none at the most external SNP #33 (Figure 5). No positive clones were obtained in DNA extracted from leaves (0/2850) at SNP#35 and #37 demonstrating that NCOs were specific to pollen DNA. The cumulative NCO frequency for both SNPs (#35 and #37) (1/203, 0.50% (CI: 0.30–0.82)) was similar to the overall CO frequency estimated with the pollen typing approach suggesting that this hotspot is equally prone to produce NCOs and COs (0.55%).

NCO events at both sites were all restricted to a single polymorphism, either #35 or #37, i.e. without co-conversion of left and/or right flanking markers, which are located 111 bp and 482 bp away for #35 and 166 bp and 340 bp for #37 (Figure 5). Thus, the mean minimal tract was 1 bp if only the polymorphism converted was considered and, the mean maximal tract was 552 bp if the tract was extended to either side just before the next non-converted polymorphism (276 bp when the minimal and maximal mean are averaged).

We recovered unequal numbers of NCOs in both directions: two ‘LtoCtoL’ and six ‘CtoLtoC’ were detected at polymorphism #35 while one ‘LtoCtoL’ and six ‘CtoLtoC’ at polymorphism #37 (Figure 5). When all NCOs were pooled, the difference between NCO rates in reciprocal orientations (‘LtoCtoL’ versus ‘CtoLtoC’) was significant (*p-val* = 0.018). This result strengthens the hypothesis that initiation occurs preferentially on the L*er* allele at the 14a1 hotspot (see above).

**Figure 6:**
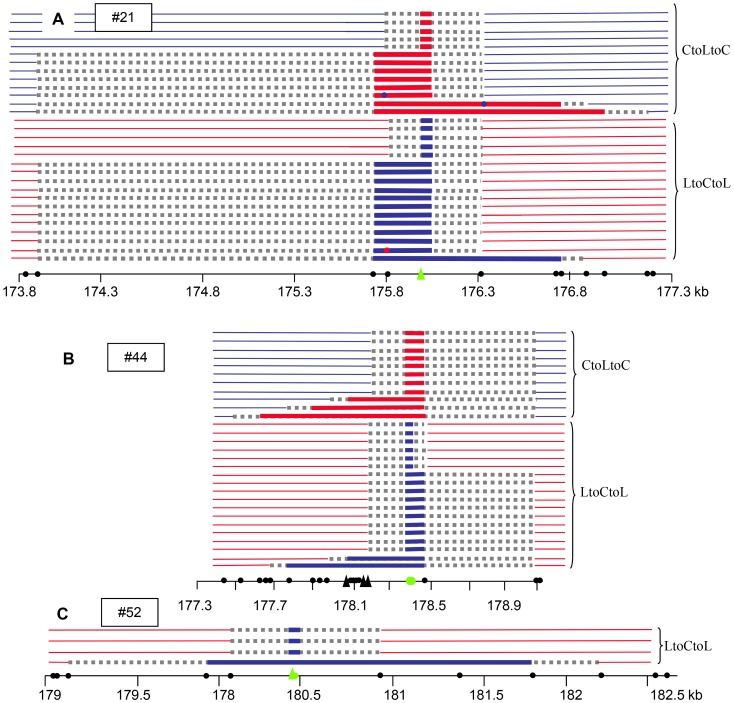
NCO events at 130×. (A) SNP #44, (B) SNP #21, (C) SNP #52. The positions of the SNPs genotyped are indicated by green filled circles (SNPs) or triangles (indels). The polymorphisms are indicated by filled black circles along the chromosome coordinate axes. Triangles: insertion or deletion greater than 7 bp. Thick red or blue horizontal lines: converted SNPs, Thick grey dotted horizontal lines: interval in which NCO tract ends are located. Blue: Col; Red: L*er*.

At 130×, NCO molecules were characterized using a PCR-based “pollen-typing” strategy (see Materials and Methods; Figure 1; [30]). Allele-specific PCR was performed using either Col specific or L*er* specific primers on 96 samples, each containing 4,145 F1 pollen genomes or 4,800 F1 leaf genomes. Then, to specifically detect NCO molecules, allele-specific PCR was carried out at three different SNPs (Figure 1; see Material and Methods; [30]): the three polymorphic sites #21, #44 and #52 were 2339 and 2052 bp away, respectively (see green dots in Figure 3B). SNP#44 is next to the center of the hotspot where the CO frequency is maximal, #21 is located in the left section of 130× where the CO rate is low and #52 is to the right where CO rates were average. For the control, DNA extracted from leaves (almost 468,000 genomes), no PCR product was amplified at SNP #44. In DNA extracted from pollen (almost 398,000 genomes), 29 NCO events were found at SNP#44 (Figure 6A) demonstrating that NCOs were specific to pollen DNA. Thirty and four NCO events were found at SNP#21 and #52, respectively (Figure 6B, 6C). The observed NCO frequency was approximately 0.007% (CI: 0.005–0.010), 0.008% (CI: 0.005–0.011) and 0.001% (CI: 0.0004–0.0033) at #44, #21 and #52, respectively. When the results obtained at the three SNPs were pooled, the NCO rate is 0.017%, which is roughly thirty times less than the overall CO frequency (0.53%).

At SNP #44, 23/29 NCO tracts extended to the right toward the neighboring polymorphism (89 bp away), while polymorphisms to the left were co-converted in only five tracts, but over a greater distance (up to 791 bp) (Figure 6B); similarly, at #21, 20/30 NCOs included the first two polymorphisms on the left (275 bp) whereas only five extended to the right but again over a greater distance (up to 1028 bp) (Figure 6A). This apparent non-symmetrical distribution of the NCO tracks reflects the asymmetrical scattering of the SNPs on either side of #44 and #21. At both sites, numerous SNPs are present only on one side, leading to an accurate analysis of the breakpoints whereas on the other side only distant SNPs were available. The minimal track length means were comparable for both SNPs (160 bp at #21 and 278 bp at #44) whereas the maximal mean track length was more than three times longer at #21 (1798 bp) when compared to #44 (492 bp). The longest NCO track was found at #52: six SNPs were co-converted along a tract of 1882 bp that could extend up to 3045 bp. Interestingly, none of the NCO tracks covered either #21 and #44 or #44 and #52. At #21 three NCO tracts were chimeric (with more than two exchange points): two ‘CtoLtoCtoLtoC’ and one ‘LtoCtoLtoCtoL’ (Figure 6A).

We also noticed that as observed for 14a1, an excess of ‘LtoCtoL’ (36) NCOs compared to ‘CtoLtoC’ (23) NCOs were detected at both #21 and #44. At #52, only ‘LtoCtoL’ NCOs were obtained. In the latter case, it was not possible to determine whether there was an absence of ‘CtoLtoC’ NCOs in our starting pool of almost 398000 genomes or if these were missed by pollen-typing. However, when the results were pooled for #21 and #44, the difference was not significant (‘LtoCtoL’: 0.009% (CI: 0.007–0.013); ‘CtoLtoC’: 0.006% (CI: 0.004–0.009)).

### CO and NCO rates in the *Atmsh4* mutant

**Figure 7:**
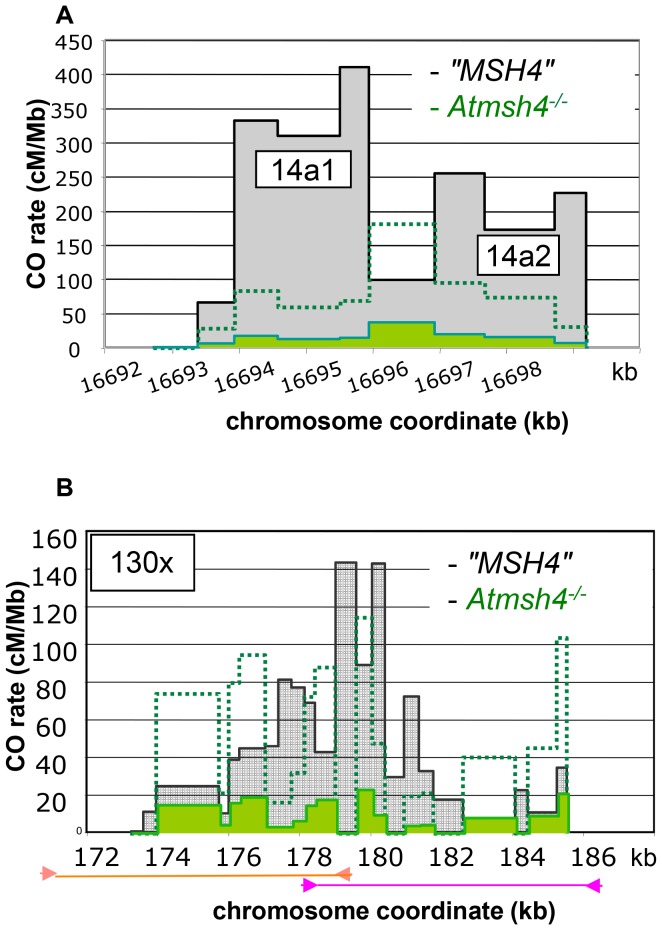
Distribution of CO rates across the 14a (A) and 130× (B) hotspots in *Atmsh4^−/−^*. (A) One hundred and eight and 48 COs were analyzed in “*MSH4*” and *Atmsh4* ^−/−^ respectively. Grey distribution: “*MSH4*”. Green distribution: *Atmsh4^−/−^*. Green dotted line: 5× magnification of the *Atmsh4* ^−/−^ distribution. (B) Two hundred and forty five and 58 COs were analyzed in “*MSH4*” and *Atmsh4* ^−/−^ respectively. Grey distribution: “*MSH4*”. Green distribution: *Atmsh4* ^−/−.^ The localization of the two overlapping PCRs is shown with orange (left part) and pink lines (right part). Green dotted line: 5× magnification of the *Atmsh4* ^−/−^ distribution.

In *A. thaliana*, on the basis of chiasma counts in mutant backgrounds, it is assumed that 85% of COs belong to the interference dependent pathway (class I), while the remaining 15% are interference-free (class II) [32]. To test the contribution of both CO pathways at the 130× and 14a hotspots, we analyzed CO rate and distribution in an *atmsh4* mutant background in which interfering COs are absent. Crosses were made between hemizygous Col and L*er* lines containing a T-DNA insertion in the *AtMSH4* gene (see Material and Methods). Meiosis appeared regular in both *AtMSH4* ^+/−^ Col and L*er* parents and the F1s *AtMSH4* ^+/+^ or *AtMSH4* ^+/−^. Meiosis, however, was disturbed in the F1 *Atmsh4* ^−/−^ with a dramatic reduction in chiasma number as described in (Higgins et al.2004; data not shown). We set up pools of *Atmsh4* ^−/−^ or *AtMSH4* ^+/−^ or *AtMSH4* ^+/+^ F1 plants, extracted gDNA from their pollen and performed pollen-typing PCR to detect CO molecules. CO rates were not statistically different in *AtMSH4* ^+/+^ and *AtMSH4* ^+/−^ at either 14a or 130× (data not shown). Thus pollen gDNA from *AtMSH4* ^+/+^ and *AtMSH4* ^+/−^ was pooled and is referred to as “*MSH4*” in the following experiments. As expected, in the *Atmsh4* ^−/−^ pollen, when we conducted the experiment at the 14a locus, we detected a dramatic decrease (12 fold) in CO frequency compared to the “*MSH4*” CO rate (Table 1). However, this frequency is likely to be slightly over-represented because the proportion of viable pollen grains depends on the number of bivalents (i.e. pairs of homologous chromosomes containing a CO). We then analyzed CO distribution (Figure 7A). Surprisingly, the two hotspots, 14a1 and 14a2, were affected differently by the mutation. In “*MSH4*”, the majority of COs (61%) occurred in 14a1 (ratio 14a1/14a2: 1.6). At *contrario* in *Atmsh4* ^−/−^, the proportion of COs between 14a1 and 14a2 was inversed (ratio 14a1/14a2: 0.5; chi2 *p-value* = 8.7 10^−5^) (Figure 7A; Table 2). At 130×, we performed two series of overlapping PCRs to cover the whole area (see Material and Methods; Figure 7B). We also obtained a lower rate of CO frequency in *Atmsh4* ^−/−^, but at a different level in the left (13 times lower), and right (78 times lower) sections of the loci (Figure 7B; Table 1).

**Table 1:** CO rates in *Atmsh4* at 130× and 14a.

	*“MSH4”*	*Atmsh4^−/−^*	R
**14a**	1.27% (0.73–2.08)	0.11% (0.06–0.18)	12
**130× left**	0.67% (0.32–1.21)	0.05% (0.04–0.06)	13
**130× right**	1.56% (0.88–2.62)	0.02% (0.01–0.1)	78

Left and right refers to the two overlapping distributions used to detect and map all COs at 130× (see Material and Methods). Confidence intervals are shown in brackets. R is the ratio of CO rates obtained in “*MSH4*” on *Atmsh4^−/−^*.

**Table 2:** Comparison of CO partitioning in 14a1 and 14a2.

	*“MSH4”*	*Atmsh4^−/−^*
**14a1**	61%	39%
**14a2**	27%	73%

The difference between the numbers of COs observed in 14a1 and in 14a2 between “*MSH4*” and *Atmsh4* ^−/−^ is highly significant using a chi-square test: *p-value* = 8.7×10^−5^.

Next, we tested the NCO frequency in the *Atmsh4* mutant background and “*MSH4*” at both loci. At the SNP#35 in the center of 14a1, 41 NCOs were detected and confirmed among 11,896 colonies in pollen DNA extracted from *Atmsh4* ^−/−^ and 20 among 3,600 colonies for “*MSH4*” pollen DNA. Thus, at this marker the NCO rate was comparable in both genetic backgrounds (Table 3). At 130×, in the *Atmsh4* ^−/−^ pollen DNA, among 83,656 molecules, two NCOs were detected at #44, which gave a NCO frequency of 0.0024% (CI: 0.00074–0.0086). In “*MSH4*”, the NCO frequency (84 events/545844 genomes) was significantly higher at the same SNP 0.015% (0.012–0.019) (*p-value* = 0.0035) (Table 3; Figure S3). Therefore, the NCO frequency at #44 decreased considerably (six fold) in the absence of MSH4.

**Table 3:** NCO rates in *“MSH4” and Atmsh4^−/^−* at 130× and 14a1.

	*“MSH4”*	*Atmsh4^−/−^*	R
**14a1-SNP35**	0.55% (0.36–0.86)	0.34% (0.25–0.47)	1.6
**130×-SNP44**	0.015% (0.012–0.019)*	0.0024% (0.0007–0.0086)*	6.2

Confidence intervals are shown in brackets. R is the ratio of NCO rates obtained in “*MSH4*” on *Atmsh4* ^−/−^.

* *p-value* = 0.0035.

### Hotspot strength and landscape vary depending on the genetic background

**Figure 8:**
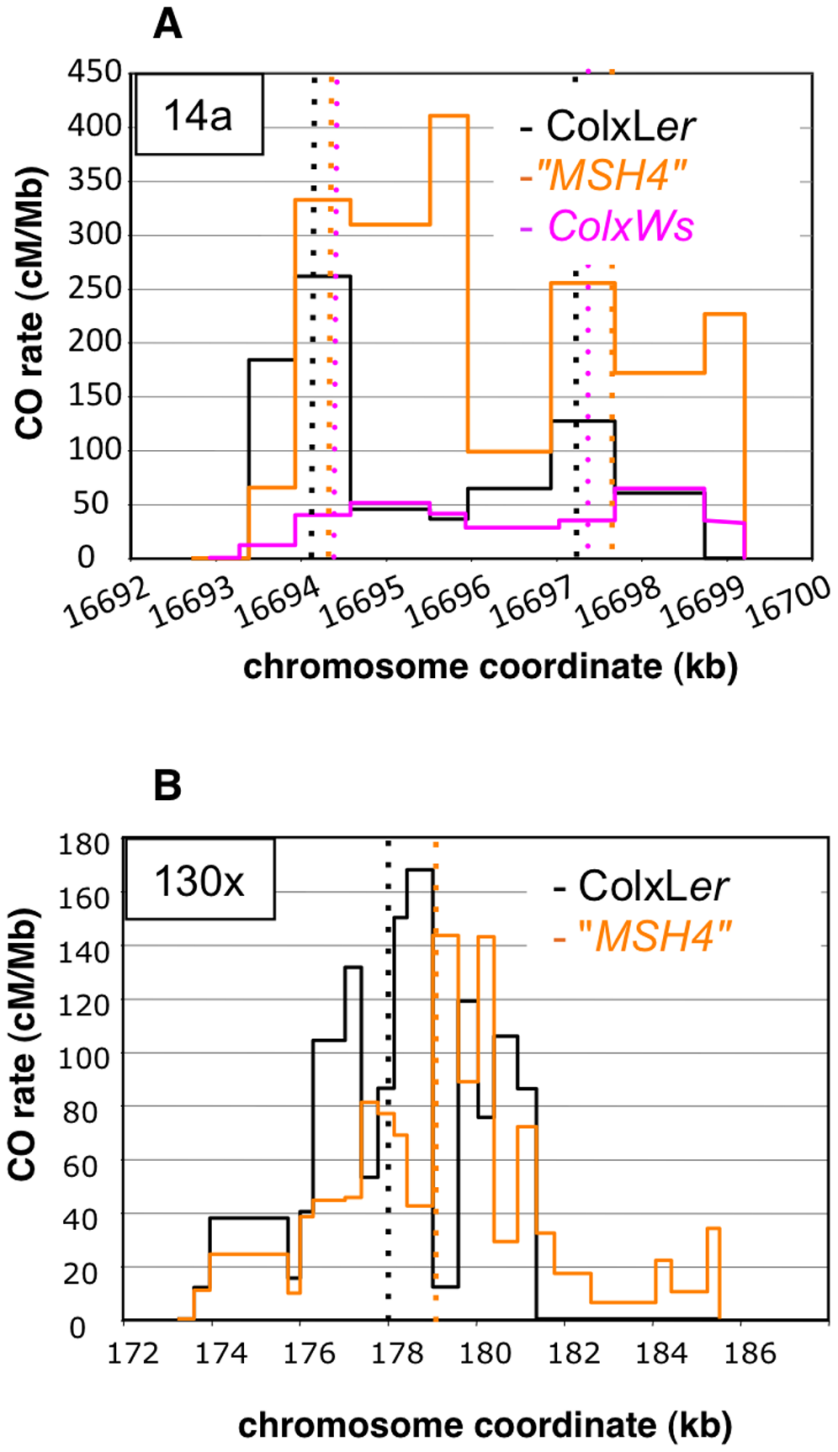
Distribution of CO rates across the 14a (A) and 130× (B) hotspots in different genetic backgrounds. (A). One hundred and four, 108 and 87 COs were analyzed in ColxL*er* (black), “*MSH4*” (orange) and ColxWs (purple), respectively. Thin dotted vertical lines: median position at 14a1 and 14a2 for each distribution. (B). One hundred and sixty seven and 245 COs were analyzed in ColxL*er* and “*MSH4*”, respectively. Thin dotted vertical lines: median position for each distribution. Using a Fisher exact test, the two distributions were found to be highly significantly different (*p-value* = 2.2×10^−16^).

We selected two other *Arabidopsis* accessions for which we could use the same allele-specific primers to perform pollen typing but which have different levels of polymorphisms within the DNA sequence at the 14a hotspot: Pyl-1 (8AV) and Ws-4 (530AV), (Material and Methods). Between Col and the three accessions L*er*, Pyl1 and Ws-4, there are 0.43%, 0.53% and 0.63% of polymorphisms distributed along the 5 kb of the 14a hotspot (Figure S1). We also included the “*MSH4*” data in this study because it is another ColxL*er* F1with exactly the same sequence at both the 14a and 130× loci. We observed considerable variation in CO rates at the 14a loci. Strikingly, the 14a hotspots almost disappeared in ColxPyl-1. There were 100 times less COs than in “*MSH4*”, 60 times less than in ColxL*er* and 23 times less than in ColxWs and even 12 times less than in the mutant *Atmsh4* ^−/−^ background (Table 4; Figure 8A). In ColxWs, the CO rate (0.21%) was in the same range as in ColxL*er* (0.55%) but significantly less than in “MSH4” (1.27%; Table 4).

**Table 4:** CO rates at 14a and 130× in various genetic backgrounds.

	ColxLer	“*MSH4*”	ColxWs	ColxPyl1
**14a**	0.55% (0.29–0.95)£	1.27% (0.73–2.08)* +	0.21% (0.09–0.42)*	0.009% (0.005–0.02)£ +
**130× left**	0.25% (0.13–0.41)	0.67% (0.32–1.21)	ND	ND
**130× right**	0.17% (0.09–0.27)°	1.56% (0.88–2.62)°	ND	ND

Left and right refers to the two overlapping distribution used to detect and map all COs at 130× (see Material and Methods). Confidence intervals are shown between brackets.

* *p-value* = 0.027;

+ *p-value* = 0.027 ;

£ *p-value* = 0.0068;

° *p-value* = 0.007.

Surprisingly, we observed that the CO and NCO rates and CO distribution at 130× differed significantly between the two ColxL*er* F1s used in this study (ColxL*er* and “*MSH4*”), whereas no significant variation was obtained at 14a (Table 4; Figure 8). We also observed another difference, with ‘LtoC’ and ‘CtoL’ exchanges peaking in the same interval in ColxWs and in “*MSH4*” but distant in ColxL*er* (see above) (Figure S2). Moreover in “*MSH4*” we recovered a comparable number of ‘LtoCtoC’ and ‘CtoLtoC’ NCOs (Figure S3). Thus the bias in recombination appears to only exist in ColxL*er*. We believe that the differences in CO rates and distribution between these two F1s are robust, as they were observed in several different experiments (data not shown) but as mentioned above the hotspot sequences are identical in these two lines.

## Discussion

### Clusters of COs at meiotic recombination hotspots

Here, we characterized several meiotic recombination hotspots on *A. thaliana* chromosome 4. Both the rate and distribution of COs and the occurrence of NCOs across these regions confirm that they are indeed true meiotic recombination hotspots.

At 14a, CO distribution patterns fit with the existence of two independent hotspots located very close to each other. 14a1 has a very high peak rate of COs and is narrow (1,475 bp) while 14a2 is broader (3,775 bp) and peaks at less than half the rate of 14a1. At 130×, the CO landscape is more complex: the CO distribution is broad and does not conform to a single Gaussian curve. Instead, it is irregular with alternating peaks and valleys. In this region, COs may originate from a single initiation zone: the irregularities observed in the distribution pattern could be explained by the presence of several insertions/deletions - which are 20, 13, 12, 70, 10, 7, 11 and, 3 bp wide respectively - along the region (Figure 3B; Figure 6). Such heterologies could either block branch migration of double Holliday junctions or channel recombination intermediates towards NCOs or exchanges between sister chromatids, as suggested previously for the mouse *HS22* hotspot [33]. However, even if at 130×, heterologies could result in a slight drop in CO rate, there is not a dearth of COs as described in several mammalian hotspots [6], [10], [34], [35]. In fact, the RuvAB branch migration helicase has been shown to bypass 1000 bp heterologies *in vitro* [36]. Alternatively, 130× could be, like 14a, a cluster of several close hotspots (three or more), each which derived from a discrete initiation zone and the resulting CO distributions could overlap extensively. This last hypothesis is strengthened by the fact that NCOs are initiated independently in at least three regions around SNPs #21, #44 and #52 and the conversion tracks do not overlap between these three regions (Figure 6).

Interestingly, in maize, there are two regions where the fine-scale distribution of COs has been characterized (the *a1* [37], [38] and bronze (*bz*) regions [39], [40]). At *a1* the pattern is similar to that of 130× with COs distributed throughout a wide region (10 kb) with peaks and valleys but with no region devoid of COs between two peaks. At *bz*, there are at least three sections where hotspots have been detected in a 99 kb region but based on the data provided whether each section is itself a cluster of hotspots or a unique hotspot could not be determined [39], [40]. The occurrence of hotspot clusters (two or more) within less than 12 kb was also described in at least four regions in human: DNA1-DNA2-DNA3, DMB1-DMB2 [41], *NID2a-NID2b*, *MSTM1a-MSTM1b* [34]. We can therefore hypothesize that in *Arabidopsis* there are regions of tens of kb that are permissive for recombination and that within these regions, recombination hotspots arise at sites where particular sequence motifs and/or chromatin modifications target the activity of Spo11 [42]–[46].

The center of all three of the hotspots described here lies close to gene promoters, which are active in *A. thaliana* meiocytes in both Col and L*er* (three to four times higher than the average transcription level, [47]). In the other *Arabidopsis* hotspot described recently, *3a*, the apparent CO peak lies in a short intergenic region where transcription terminates for both genes [48]. In maize, the majority of characterized hotspots are localized in genes [40], [49] but there was not sufficient resolution to determine if the CO peak lies within the promoter region. Most DSB hotspots in *S. cerevisiae* [50]–[52] coincide with promoter regions while in *S. pombe*, hotspots lie preferentially in large intergenic regions [53]. In human and mice recombination activity occurs near genes but away from transcription start sites (TSS) [54]. However, even if the localization of meiotic recombination hotspots seems drastically different in *S. cerevisiae* and in mice, the underlying mechanisms may not be so divergent. In *S. cerevisiae*, meiotic DNA DSBs are formed in nucleosome depleted regions enriched in histone H3 trimethylated on lysine 4 (H3K4me3) [55]. In mice, an enrichment of H3K4me3 is also detected at DSBs sites [56]. Recently, a genome-wide correlation between recombination sites, the insertion sites of the transposable element *Mu* and the chromatin modification H3K4me3 was also reported in maize. As *Mu* insertion sites also correlate strongly with recombination sites, it was suggested that the local chromatin structure could play a key role in both mechanisms [57]. Others chromatin modifications have been shown to be associated with meiotic recombination hotspots in others species. Acetylation of lysine 9 of histone H3 in *S. pombe* [46] and levels of H3K9m3 and H2AK5ac in *C. elegans* modulate meiotic DSB formation [58], [59]. In *A. thaliana*, the *3a* and 14a hotspots but not 130× lie in a low nucleosome density region [48] (this study). It is important to note that the nucleosome data we are comparing to was generated from somatic (seedling) tissues and we therefore cannot rule out that nucleosome occupancy may differ during meiosis. However, nucleosome occupancy in yeast and mammals is similar between meiotic and mitotic cells [60]–[62]. Thus it could be that several different chromatin states act on the localization of meiotic DSBs. The potential similarity between the location of *S. cerevisiae* and *Arabidopsis* hotspots in promoter regions could be due to the resemblance of their genomic structures. Both are compact, have a high density of genes along chromosomes and small intergenic regions [63], [64].

### Variation in CO distribution

We measured the CO rate and distribution at 14a during meiosis of four different F1s. In ColxWs, the CO rate was significantly different to “*MSH4*” but even more strikingly, the hotspot almost disappeared in ColxPyl1. There were even less COs than in the *Atmsh4*−/− background. Both Ws and Pyl1 exhibit differences at the DNA sequence level compared to Col (0.53% and 0.63% respectively; Figure S1) that could explain this variation. A correlation between a decrease in the rate of meiotic recombination and polymorphism level has been reported in various species including plants and it has been shown that the mismatch repair machinery is involved in this drop in meiotic recombination rate [65]. Transposon insertion has also been shown to modify meiotic recombination at *a1* [37] and *bz* [40] in maize. Alternatively, the disappearance of the hotspot in the ColxPyl1 cross could be due to a modification of a sequence inside 14a, crucial for the initiation of recombination. Similar results have been reported in mice and human where a mutation in the recognition site of PRDM9 can dramatically influence the hotness of a hotspot [66], [67]. Thus a combination of factors acting in “*cis*” or “*trans*” could influence the behavior of the 14a loci during meiosis.

Although the 8.8 kb sequence of the 14a locus in the two other F1s, ColxL*er* and “*MSH4*”, is identical, significant differences were observed at their hotspots. In ColxL*er* a bias in CO distribution and directionality of NCOs suggests that preferential initiation on the L*er* chromosome occurred but this was not observed in “*MSH4”*. At 130×, which is also identical in both F1s, we also observed significant differences in CO rate and distribution between ColxL*er* and *“MSH4”* but the NCOs were the same. The accessions used to obtained these two F1s are clearly related to each other (http://arabidopsis.info/protocols/ler.html) but nevertheless they have evolved since they were isolated and it could be that some key mutations were selected that changed in “trans the local behavior of the region. Alternatively, the epigenetic status of the two F1s may have changed leading to differences in the activity of hotspots.

### Meiotic NCOs

We isolated and characterize dozens of meiotic NCOs at two loci for the first time in *Arabidopsis thaliana*. Three recent studies addressed the detection and rate of meiotic NCOs in *A. thaliana*. One recorded gene conversion events associated or not with COs at seven loci but only one NCO was detected at one locus [18]. The two other studies used NGS for genome-wide detection of COs and NCOs. These two studies gave contradictory results on the rate of NCOs. One estimated NCOs to be rare meiotic events (on average two per meiosis) [16] whereas the other one predicted up to 3000 NCOs per meiosis [17]. However, in this latter study, up to 30 to 40 COs per meiosis were also predicted which is three to four times higher and not consistent with numerous genetics or cytological studies performed with wild type crosses, suggesting a large over estimation of NCO rates in this study. Our data clearly show that NCO rates are highly unhomogeneous between hotspots. At 130×, NCOs were detected at three polymorphic sites distributed along the hotspot with an overall rate of 0.016%, thus 30 times less than that of COs. At 14a, the observed NCO rate was similar to that of COs (0.5%). In *A. thaliana*, meiotic DSBs sites have been estimated at between 100 and 200 per meiotic cell based on the number of RAD51 or DMC1 foci (two DSB repair proteins) at mid-prophase [12]–[14]. The proportion of these DSBs repaired on the sister or homologous chromatids is unknown, but if only half of them are repaired as NCOs, there should be five to ten times more NCOs than COs. However, neither the NGS genome-wide data [16] or our data at two hotspots support this. We propose that NCOs are very small and in most cases are not detectable because they do not convert a SNP. Indeed, most of the NCO tracks detected in this study were single SNPs.

We also detected three NCO events with a discontinuous conversion pattern: two ‘CtoLtoCtoLtoC’ and one ‘LtoCtoLtoCtoL’ (Figure 6A). In all cases, the discontinuity was related to a single SNP (A/T or C/A). Similar complex conversion events have been detected in other species [68]–[70]. It was suggested that chimeras could result from template switches between non-sister and sister-chromatids during DSB repair [68], [70]. Alternatively, at this particular locus, the mismatch repair machinery may fail to convert all mismatches contained in the heteroduplex generated by the homologous recombination machinery.

### MSH4 plays a key role in CO and in NCO formation but with a different strength depending on hotspots

According to the current view of meiotic DSB repair (reviewed in [71]), in most species (including *S. cerevisiae*, mammals and plants) most if not all NCO events arise through a “Synthesis Dependant Strand Annealing” (SDSA) mechanism whereas COs are formed by two distinct pathways, which generate either interfering COs (class I) or non-interfering COs (class II). MSH4 belongs to a group of highly conserved proteins, called ZMM, that are essential for the “class I” CO pathway [19]. In *S. cerevisiae* (reviewed in [19]), and *Sordaria macrospora* [72], the mutation of *MSH4* leads to a pronounced decrease in CO number. Therefore, in *Arabidopis Atmsh4*, the decrease in COs that we observed at 130× and 14a was expected but the change in CO distribution was more surprising. Thus, 130× and 14a are very likely to be clusters of hotspots and the proportion of *MSH4* dependent COs appears to vary markedly between hotspots within the cluster and between hotspots.

Surprisingly, we also found that in the *Atmsh4* mutant at 130× there is a six fold decrease in the frequency of NCO events. In *A. thaliana*, mice and *S. macrospora*, the MSH4 protein is localized in numerous foci along chromosome axes as early as mid-late leptotene and then the number of foci decreases to zero at the end of pachytene [32], [72], [73]. In these three species, however, the maximum number of MSH4 foci far exceeds the number of COs. Furthermore, in *S. macrospora* MSH4 is present at virtually all sites of interaction between homologs (COs and NCOs) at the onset of zygotene, where it appears to play a role in the orderly progression of the pairing and synapsis processes [72]. We now propose that in all eukaryotes, beyond its role in class I CO formation, MSH4 is involved in the formation or stabilization of at least some of the recombination intermediates leading to NCOs.

In conclusion, we have formally demonstrated that true meiotic recombination hotspots exist in the plant *Arabidopsis thaliana*. We have also established that COs and NCOs occur at very high rates at these hotspots, as observed in yeast and mammals. However, we have shown that the pattern of COs and NCOs differs from that described in other species. There is therefore a need for the analysis of more hotspots in a diverse range of species in order to understand the underlying mechanisms that control them.

## Materials and Methods

### Plant material

The *Arabidopsis thaliana* accessions “Columbia-0” (Col, 186AV), “Landsberg *erecta*” (L*er*, 213AV), Pyla-1 (Pyl-1; 8AV) and Wassilewskija-4 (Ws-4; 530AV) were obtained from the “Centre de Ressources Biologiques” at the “Institut Jean Pierre Bourgin”, Versailles, France. The *MSH4* mutant lines SALK_136296 (Col-0) [74] and CSHL_GT14269 (L*er*) [75] were provided by NASC (http://nasc.nott.ac.uk/). All plants were grown in the greenhouse under standard conditions.

After crossing, we used PCR screening to select homozygous *AtMSH4* ^+/+,^^+/−^ and ^−/−^ plants among the F1. The oligonucleotides used were: (i) N636296U CTTCTTGCAGGTTfGTGTTTG - N636296L GCCAGCTGTTTTTGTTGTC and N636296L - LbSalk2 TCCCGCTCAGAAGAACTC to genotype the wild type and the mutant allele respectively in Col (ii) GT14269U CCGTTCAAATGTTTGCCATAC - GT14269L TTTCACCTTCCTAACGGTGC and CSHLds5-4 TACGATAACGGTCGGTACGG - GT14269L to genotype the wild type and the mutant allele respectively in L*er.*

Characterization of male meiosis by cytology was carried out as described in [76]. Chiasma counts, performed on ColxL*er* and “MSH4” as described in [15], showed similar numbers of chiasmata in the two F1s (Figure S4).

### Extraction of pollen genomic DNA

Genomic DNA from pollen was extracted as described in [24]. Briefly, whole inflorescences from hybrid plants were harvested in 10% saccharose, and crushed in a “Waring Blender” (two 4 sec pulses at full speed). The homogenate, containing intact microspores and pollen grains, was then filtered and stored at −20°C until DNA extraction. Pollen grains and microspores were resuspended and incubated with proteinase K at 65°C for three hours with gentle shaking. Then, pollen grains were disrupted by mixing with glass beads with a vortex at full speed for 1 to 3 minutes. One volume liquid phenol was added and tubes were rocked for 30 min at 4°C. After centrifugation, the supernatant was recovered and nucleic acids were precipitated with sodium acetate and ethanol. Genomic DNA was dissolved in (10 mM Tris-Cl pH8, 1 mM EDTA, 100 µg/ml RNAseA) and incubated at room temperature for 15 min. Four volumes of freshly made (5 M guanidine isothiocyanate, 50 mM Tris-Cl pH 8) were then added, and DNA was purified with DNeasy minicolumns (Qiagen ref. 69106).

### Extraction of leaf genomic DNA

Genomic DNA was extracted from young leaves as described in [77]. Four volumes of freshly made (5 M guanidine isothiocyanate, 50 mM Tris-Cl pH 8) were added to the extract, and DNA was purified with DNeasy minicolumns (Qiagen ref. 69106).

### Quantification of genomic DNA

Quantification of gDNAs was performed as described in [24]. Briefly, PCR reactions were performed in 20 µl of buffer [78] with 1 U *Taq* DNA polymerase and 0.1 U *Pfu* DNA polymerase. Whenever less than 100 pg/µl of genomic DNA was used, herring sperm DNA (Clontech) was added into the reactions (1 ng/µl). Primers (sequence and genomic coordinates) are listed in Table S1. Pairs of oligonucleotides used are listed in Table S2.

Products amplified from the gDNA extracts were quantified in a series of dilutions through two rounds of PCR, using the nested allele-specific oligonucleotides (ASOs) listed in Table S1 and Table S2. The product of the first PCR was diluted 1/1000 in the second reaction. The thermal cycling profile of the reactions was: (((92°C;2 min)((92°C;20 sec)(T_m_;30 sec)(68°C;30 sec + 45 sec/kb)))×30(68°C;90 sec/kb)(4°C;∞)). After the second PCR, the proportion of negative wells among a set of aliquot reactions was approximated by e^−m^, where ‘m’ is the mean number of DNA molecules per well in the first reaction. Parental molecules were thus quantified using ASOs all specific to either Col or L*er* DNA.

### CO detection and mapping of CO exchange points

CO molecules were amplified with primers specific to either Col or L*er* allele on one side, and L*er* or Col respectively on the other side (Figure 1). Primers (sequence and genomic coordinates) are listed in Table S1. Pairs of oligonucleotides are listed in Table S2.

For mapping CO exchange points, a series of aliquot reactions was carried out, which was predicted to contain an average of less than 0.2 CO molecules, so that more than 90% of positive reactions issued from a single CO molecule. PCR products were then sequenced in order to locate exchange points from single CO molecules.

To detect COs in “*MSH4*” and “*Atmsh4*”, two series of overlapping PCRs were performed. The two PCRs overlapped on each side of the SNP#44. The first PCR used the primers 130×0LeL1 and 130×52CoR1 or 130×0CoL1 and 130×52LeR2. Then primers 130×7LeL5 and 130×47CoR2 or 130×7CoL4-130×47LeR4 were used for the left distribution whereas for the right distribution we used 130×44LeL4 and 130×72CoR2 or 130×44CoL4 and 130×72LeR4 (Table S1). At 14a, the L*er* primers were used to detect COs in Ws and Pyl1.

### Characterization of NCO events at the 130× and the 14a1 hotspots

NCO molecules at polymorphisms #44, #21 and #52 in the 130× hotspot were detected using a PCR-based strategy adapted from [25]. The outline of this approach is described in Figure 1 [30]. An allele-specific PCR was performed using either Col specific primers or L*er* specific primers on 96 samples each containing 4,145 F1 pollen genomes for ColxLer, 5,685 for “*MSH4*” or 4,800 F1 ColxL*er* leaf genomes. Then, two sets of allele-specific PCRs were carried out in parallel at one SNP to specifically detect NCO molecules. When both left and right PCR reactions were positive at one SNP, recombinant PCRs molecules were fully sequenced to (i) confirm the NCO event and (ii) map the recombinant point (Figure 1). Pairs of primers for these experiments are listed in Table S3.

For 14a1, we followed the procedure described ([30]; see Figure 1). Twenty four PCRs were carried out on DNA extracted from F1 ColxL*er* pollen corresponding to 48,000 F1 genomes, half with the primers specific from the Col parental allele and located outside the hotspot region and the other half with the L*er* primers (Table S1 and Table S2). Then after a second role of allele-specific PCR on each pool of DNA, the PCR products were pooled. The PCR products were then digested with Bgl*II* and Xba*I* and ligated into pCRIITOPOblunt (Invitrogen) between the BamH*I* and Xba*I* unique sites, using standard procedures. The ligation products were then used to transform DH10B *E. coli* strain by electroporation. Transformed cells were spread onto LB agar plates containing 100 µg/ml carbenicillin, 0.2 mM IPTG and 40 µg/m X-gal. Following blue/white screening, individual colonies were transferred to 200 µl of LB medium containing 100 µg/ml carbenicillin in 1 ml MASTERBLOCK microplates (Greiner Bio-One ref. 780215) and grown with gentle shaking at 37°C for 16 hours. Then, 100 µl of each cell culture was transferred to 96 well V-bottom microplates and spun down at 3200×g for 10 min. Cell pellets were resuspended in 100 µl of sterile water.

Bacterial clones were then genotyped using the “Chemicon Amplifluor SNPs Genotyping System”. For this purpose, oligonucleotides either specific to each parent at polymorphisms #33, #35 and #37, or non-specific primers, were designed using the Amplifluor AssayArchitect software (Table S4).

Genotyping was then performed as described in [31]. Plasmids which appeared to contain a NCO event by genotyping were fully sequenced to precisely map the gene conversion event.

### Statistical analyses

The differences between CO distribution in reciprocal orientations at a given hotspot were tested as follows: (i) CO breakpoints located on each side of the median position were grouped separately for the two reciprocal orientations (‘CtoL’ CL and ‘LtoC’ LC), thus providing four numbers CO_CLleft_, CO_CLright_, CO_LCleft_, CO_LCright_; (ii) these numbers were grouped in a contingency table for testing the association between left/right and CL/LC classification using the two-tailed Fisher's exact test.

The difference between ‘CtoLtoC’ and ‘LtoCtoL’ NCO rates at polymorphisms #35 and #37 in the 14a1 hotspot was tested using the one tailed Fisher's exact test.

The parameters of the best fitting Gaussian distributions were calculated using an R script (Supplemental File S1), which computes the least sum of squared differences between observed and theoretical (Gaussian) integrated distributions over every interval between successive SNPs.

Estimated CO frequencies and associated confidence intervals were analyzed as follows:

Repeated PCR experiments were performed on highly diluted pollen DNA samples collected on a F1 plant obtained from a cross between two homozygous parents carrying different alleles at two marker loci. Two primer pairs were used for PCR amplification. The first pair was specific for molecules carrying the alleles of the first parent at both loci, and could thus amplify half of the non-recombinant molecules. The second primer pair was specific for molecules carrying the first parental allele at the first locus and the second parental allele at the second locus, and could then amplify half of the recombinant molecules. A PCR reaction was considered positive if the template contained at least one molecule corresponding to the primer pair used. The same initial pollen DNA sample (unknown concentration *C*) was used as the template for all experiments, but at different dilutions. An *S_i_* series of experiments (indexed with *i*) was performed with the first primer pair, and an *S_j_* series (indexed with *j*) with the second primer pair. For the *k* ^th^ series, a total of *N_k_* PCR reactions was carried out using as template the initial DNA sample diluted at the rate *D_k_*. Let us note *y_k_* as the number of reactions that *did not* produce a product.

A Bayesian inference approach was used to infer the recombination rate between the two marker loci, as well as its 95% confidence intervals.

For the first primer pair, amplifying non-recombinant molecules, the probability of no amplification in a given well follows a Poisson law and is: 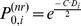
_._ The likelihood of the observed PCR results obtained with this primer pair follows a binomial law: 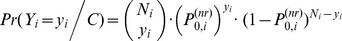


With the second primer pair, amplifying recombinant molecules, the probability of no amplification is 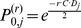 where *r* is the recombination rate between the two marker loci. The likelihood of the observed results is then: 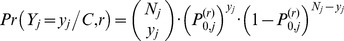


The joint likelihood of all observations (both primer pairs) is: 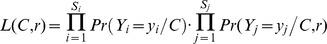


Because *C* and *r* are independent, the *a posteriori* probability of parameters *C* and *r* knowing the observations is: 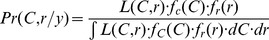 where *f_C_* and *f_r_* are the prior density functions of *C* and *r* respectively.

The prior distributions for *r* and *C* were taken uniformly distributed in [0;0.1] and 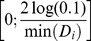 respectively, which supposes that *r*<10%, and that at least 10% positive reactions are expected with the least concentrated sample.

The *a posteriori* distributions of *r* and *C* were numerically computed by a two-dimensional scan of the parameter space (R script available upon request, and the 95% confidence intervals on *r* and *C* were determined from these distributions.

95% confidence intervals on CO and NCO frequencies were computed based on the binomial law, by numerically adjusting the frequencies corresponding to distribution function values equal to 0.025 and 0.0975. When the value of a parameter was estimated from the data, the associated 95% confidence interval is defined so there was less than 5% chance that the true value of the parameter lies outside this interval.

## Supporting Information

Figure S1 Polymorphisms at 14a in various F1. A. Localization of polymorphisms between the Col and the L*er* accessions (black diamond), the Col and the Ws-4 accession (purple triangle), the Col and the Pyl-1 accession (green dot). The numbers on the left refer to the percentage of polymorphisms between Col and the other accessions. B. CO distribution at 14 a in various F1s.
(PPT)

Figure S2 Cumulative CO rates at 14a in ColxL*er* and “MSH4.” For both reciprocal orientations, cumulated (from left to right) relative CO rates were scored at successive polymorphic sites along the region. Blue curves: cumulated relative ‘CtoL’ CO rates; dark blue: “MSH4”; light blue: ColxL*er*. Red curves: cumulated relative ‘LtoC’ CO rates; dark red: “MSH4”; light red: ColxL*er*. Vertical lines: median positions of the hotspots; dotted: “MSH4”; filled: ColxL*er*.
(PPT)

Figure S3 NCO at 130× in “MSH4” and *Atmsh4* ^−/−.^. Position of SNPs genotyped are indicated by green filled circles (SNPs) or triangles (indel). The polymorphisms are indicated by filled black circles along the chromosome coordinate axes. Triangles: insertion or deletion above 7 nt. Thick red or blue horizontal lines: converted SNPs, Thick grey dotted horizontal lines: interval in which NCO tract ends are located. Blue: Col; Red: L*er*. Yellow line: chimeric SNPs.
(PPT)

Figure S4 Chiasma number per meiosis in “MSH4” and ColxLer F1s. The number of chiasma per male meiocytes was obtained as described in [15]. Number of cells = 40 for each F1.
(PPT)

File S1 R Script to determine the parameters of the best fitting Gaussian distribution of a CO distribution.
(DOCX)

Table S1 Sequences of primers.
(DOC)

Table S2 Primers for DNA quantification and CO detection.
(DOC)

Table S3 Primers for NCO detection at 130×.
(DOC)

Table S4 Primers for NCO detection at 14a1 hotspot.
(DOC)
