# Supplementary material for: Contrasted Patterns of Crossover and Non-crossover at Arabidopsis thaliana Meiotic Recombination Hotspots
Source: PLoS Genet. 2013 Nov 14;9(11):e1003922. doi: 10.1371/journal.pgen.1003922 (PMC3828143; doi:10.1371/journal.pgen.1003922)
Supplement: Figure S1 — Polymorphisms at 14a in various F1. A. Localization of polymorphisms between the Col and the Ler accessions (black diamond), the Col and the Ws-4 accession (purple triangle), the Col and the Pyl-1 accession (green dot). The numbers on the left refer to the percentage of polymorphisms between Col and the other accessions. B. CO distribution at 14 a in various F1s. (PPT) [file pgen.1003922.s001.ppt]

## Slide 1
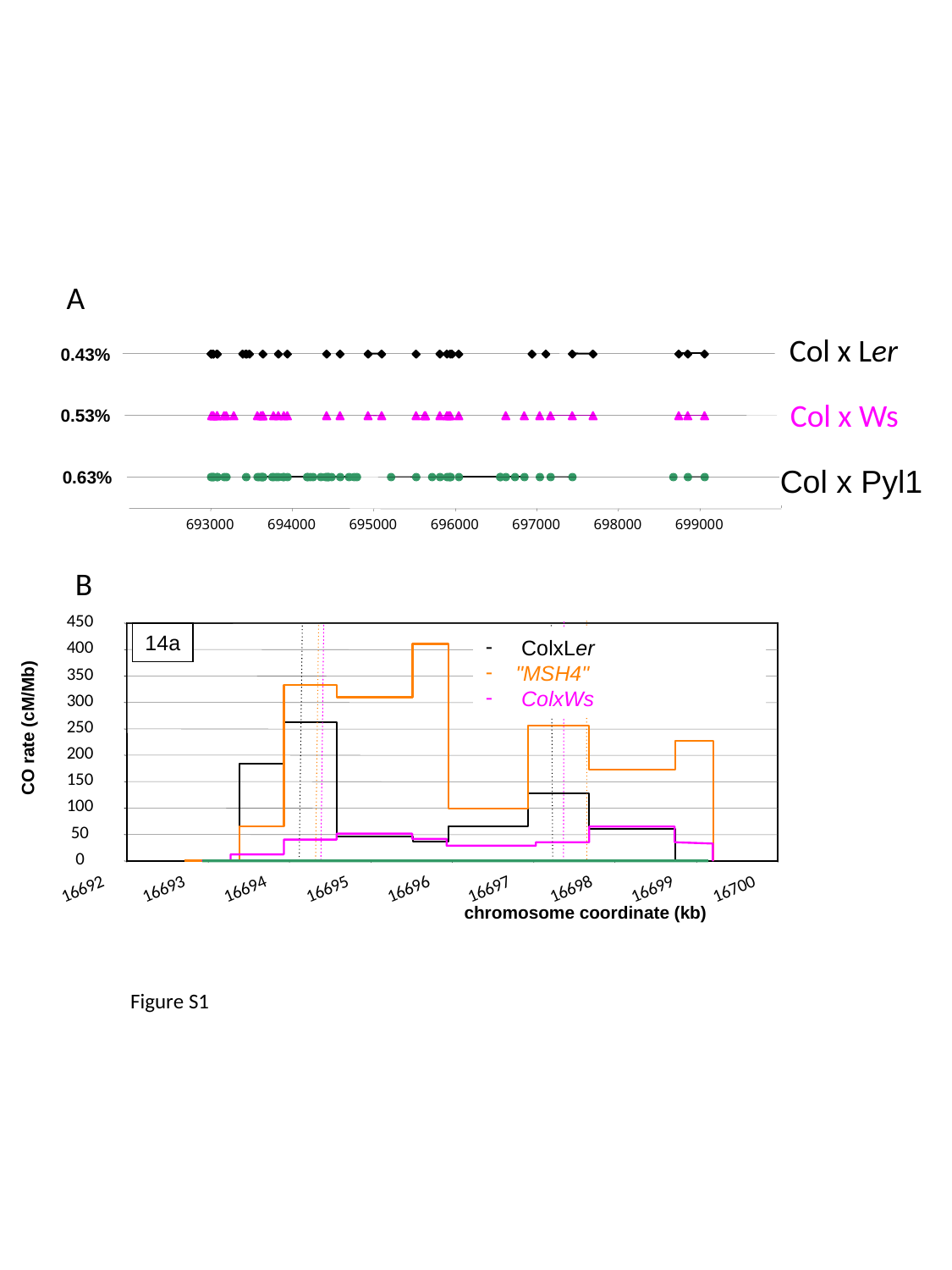

A
Col x Ler
0.43%
Col x Ws
0.53%
Col x Pyl1
0.63%
693000
694000
695000
696000
697000
698000
699000
B
450
14a
 ColxLer
"MSH4"
 ColxWs
400
350
300
250
CO rate (cM/Mb)
200
150
100
50
0
16692
16693
16695
16696
16698
16699
16697
16700
16694
chromosome coordinate (kb)
Figure S1
