# Supplementary figures and images for: Contrasted Patterns of Crossover and Non-crossover at Arabidopsis thaliana Meiotic Recombination Hotspots
Source: PLoS Genet. 2013 Nov 14;9(11):e1003922. doi: 10.1371/journal.pgen.1003922 (PMC3828143; doi:10.1371/journal.pgen.1003922)

## Slide 1
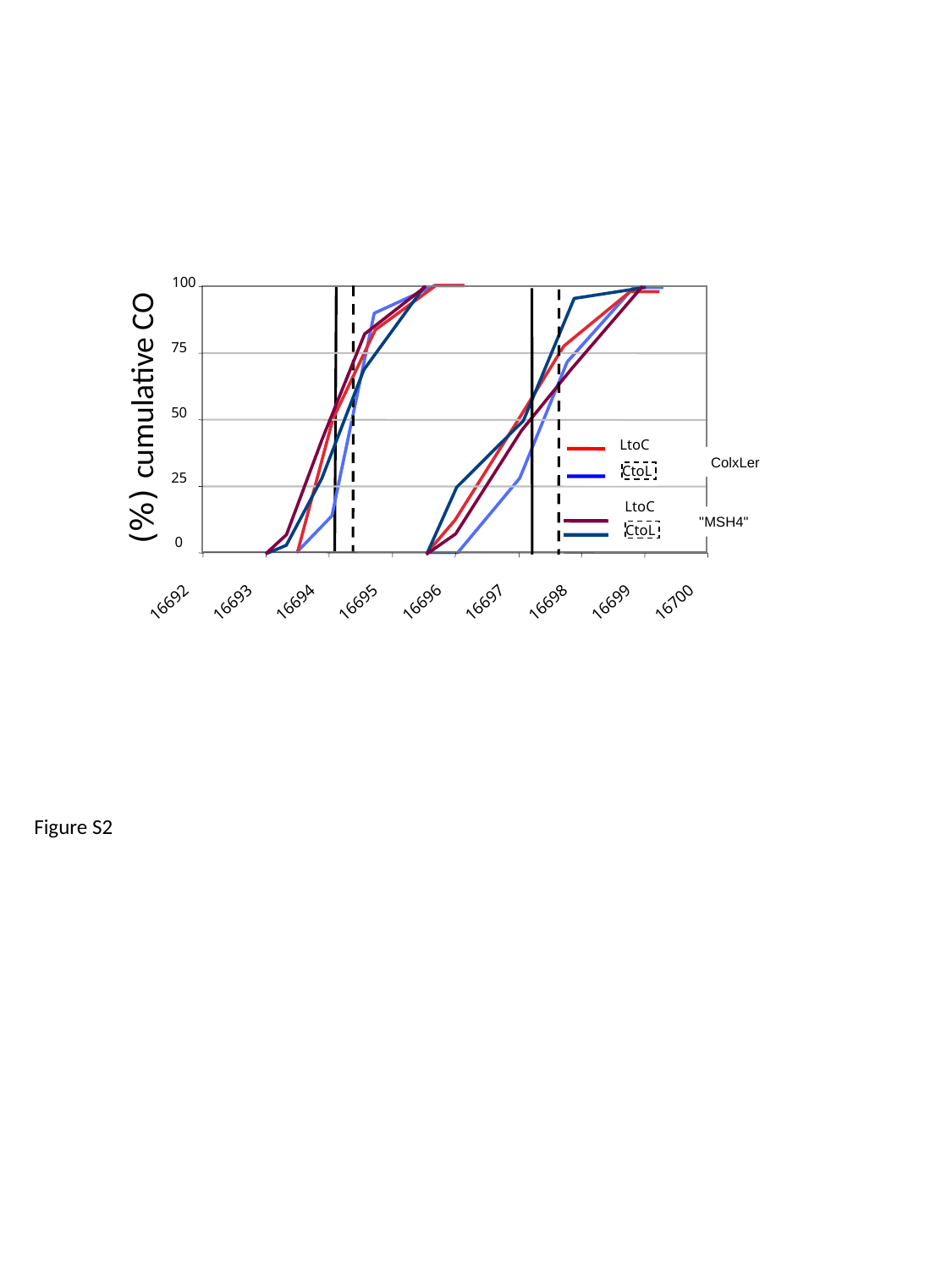

100
75
50
25
0
 cumulative CO (%)
LtoC
ColxLer
CtoL
LtoC
"MSH4"
CtoL
16699
16692
16693
16696
16698
16694
16695
16697
16700
Figure S2

Supplement: Figure S2 — Cumulative CO rates at 14a in ColxLer and “MSH4.” For both reciprocal orientations, cumulated (from left to right) relative CO rates were scored at successive polymorphic sites along the region. Blue curves: cumulated relative ‘CtoL’ CO rates; dark blue: “MSH4”; light blue: ColxLer. Red curves: cumulated relative ‘LtoC’ CO rates; dark red: “MSH4”; light red: ColxLer. Vertical lines: median positions of the hotspots; dotted: “MSH4”; filled: ColxLer. (PPT) [file pgen.1003922.s002.ppt]

## Slide 1
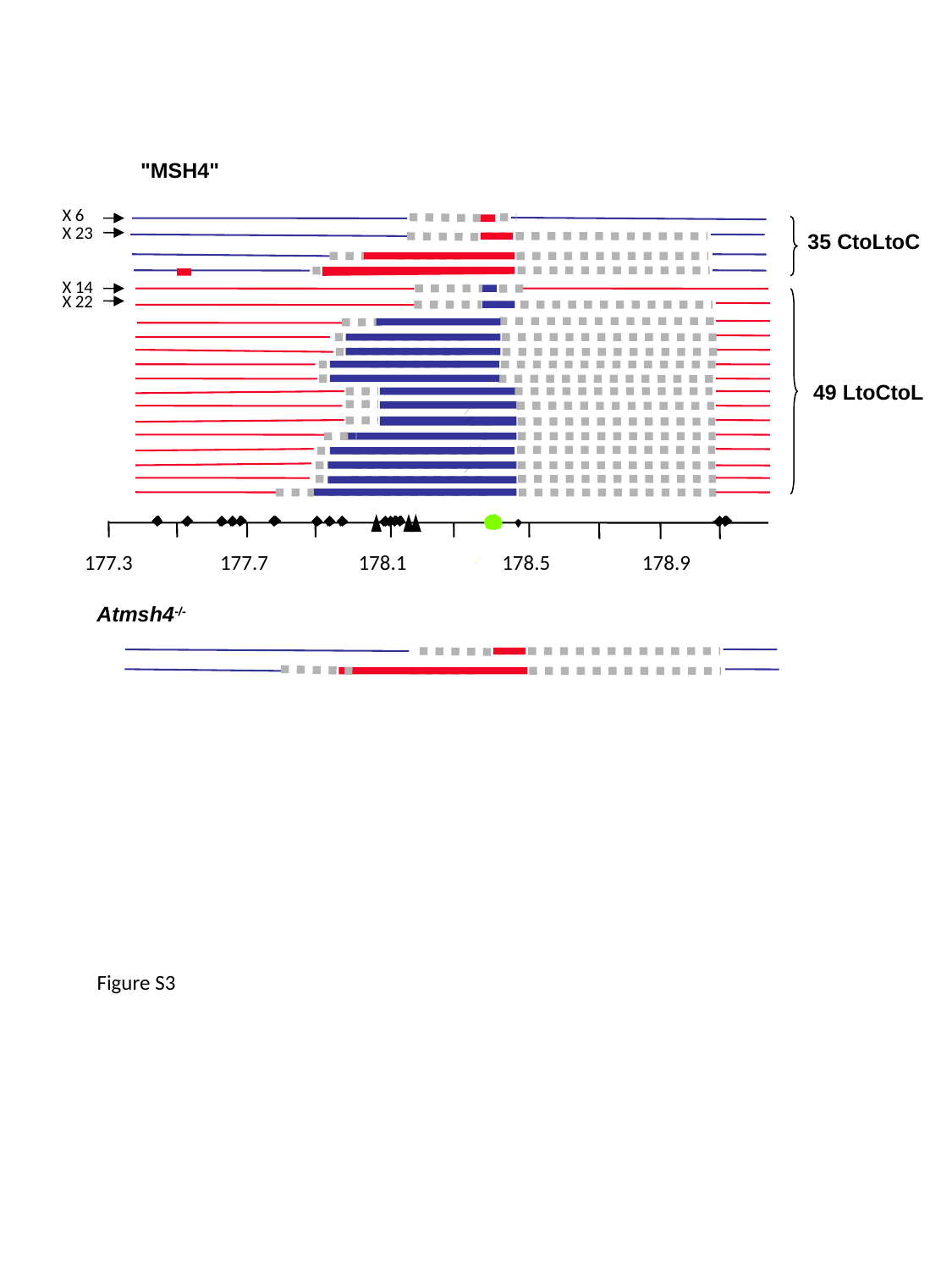

"MSH4"
X 6
X 23
35 CtoLtoC
X 14
X 22
49 LtoCtoL
177.3
177.7
178.1
178.5
178.9
Atmsh4-/-
Figure S3

Supplement: Figure S3 — NCO at 130× in “MSH4” and Atmsh4 −/−.. Position of SNPs genotyped are indicated by green filled circles (SNPs) or triangles (indel). The polymorphisms are indicated by filled black circles along the chromosome coordinate axes. Triangles: insertion or deletion above 7 nt. Thick red or blue horizontal lines: converted SNPs, Thick grey dotted horizontal lines: interval in which NCO tract ends are located. Blue: Col; Red: Ler. Yellow line: chimeric SNPs. (PPT) [file pgen.1003922.s003.ppt]

## Slide 1
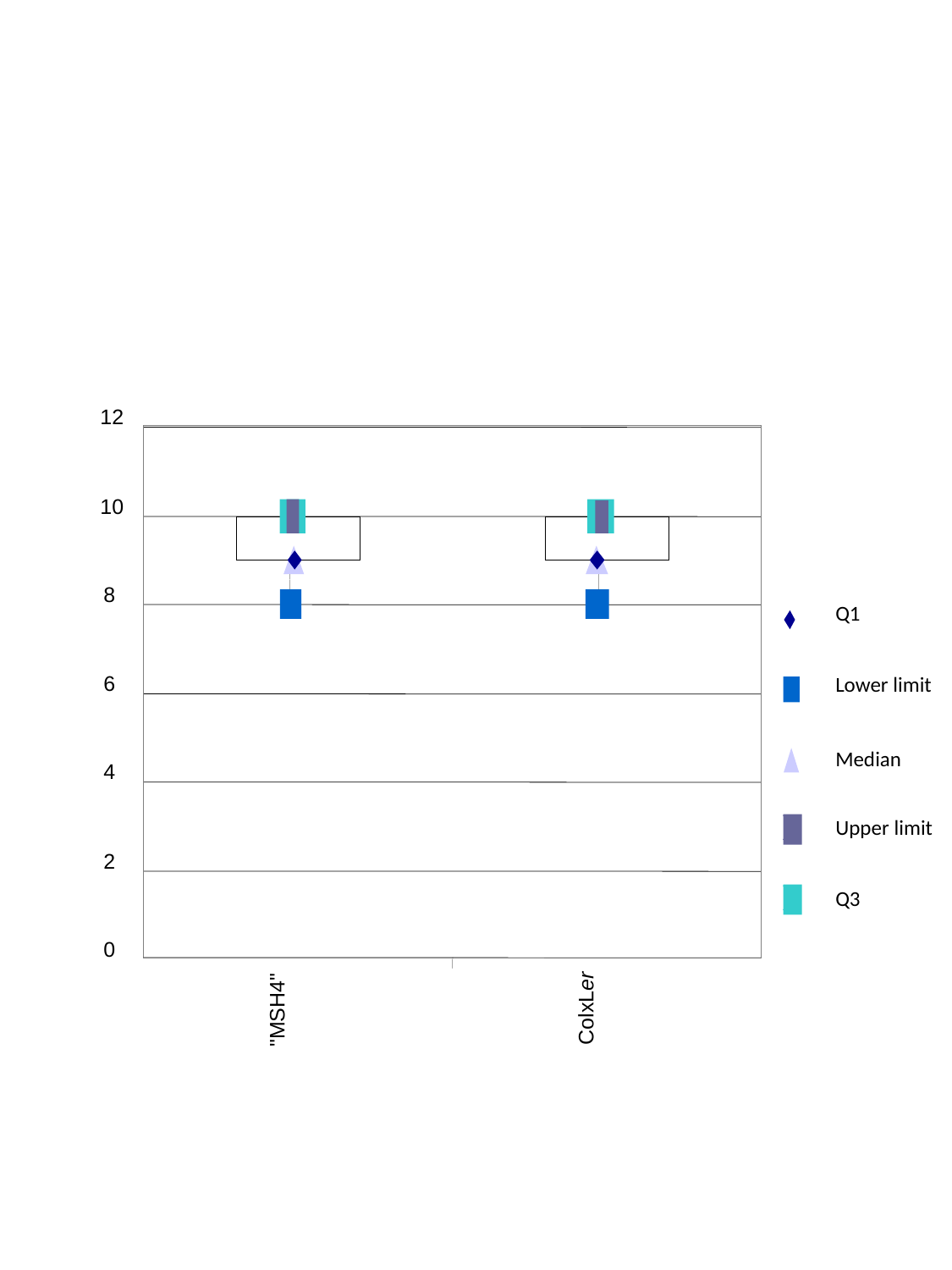

12
10
8
Q1
Lower limit
6
Median
4
Upper limit
2
Q3
0
ColxLer
"MSH4"

Supplement: Figure S4 — Chiasma number per meiosis in “MSH4” and ColxLer F1s. The number of chiasma per male meiocytes was obtained as described in [15]. Number of cells = 40 for each F1. (PPT) [file pgen.1003922.s004.ppt]
