# Supplementary material for: Contrasted Patterns of Crossover and Non-crossover at Arabidopsis thaliana Meiotic Recombination Hotspots
Source: PLoS Genet. 2013 Nov 14;9(11):e1003922. doi: 10.1371/journal.pgen.1003922 (PMC3828143; doi:10.1371/journal.pgen.1003922)
Supplement: File S1 — R Script to determine the parameters of the best fitting Gaussian distribution of a CO distribution. (DOCX) [file pgen.1003922.s005.docx]

## the file containing the CO distribution id provided as an argument to the R script

## the values must be separated by tabulations or white spaces

## the first line contains the n genomic coordinates of the markers

## the second line contains the number of COs

## the ith field corresponds to the interval delimited by the ith and (i+1)th markers

args <- commandArgs(TRUE)

exp_data_1 <- as.matrix(read.delim(file=args[1],header=FALSE,sep="\t",row.names=c("genomic_coordinate","CO_rate")))

exp_data_2 <- matrix(nrow=nrow(exp_data_1),ncol=ncol(exp_data_1)-1,dimnames=list(rownames(exp_data_1)))

n_genomic_intervals <- ncol(exp_data_2)

for (i in 1:n_genomic_intervals){exp_data_2[1,i] <- mean(exp_data_1[1,i:(i+1)])}

exp_data_2[2,] <- 100*cumsum(na.exclude(exp_data_1[2,]))/sum(na.exclude(exp_data_1[2,]))

m_lower <- exp_data_2[1,1]

m_upper <- exp_data_2[1,n_genomic_intervals]

m_range <- m_upper-m_lower

s_upper <- m_range

s_lower <- s_upper/100

s_range <- s_upper-s_lower

while (round(m_lower,6) != round(m_upper,6) || round(s_lower,6) != round(s_upper,6)){

max_dist <- 0

max_dist_i <- 1

max_dist_j <- 1

max_i <- 10

max_j <- 10

sum_of_squares <- matrix(nrow=max_i,ncol=max_j)

m_steps <- vector(length=max_i)

s_steps <- vector(length=max_j)

for (i in 1:max_i){m_steps[i] <- m_lower + (i-1)*m_range/(max(1,max_i-1))}

for (j in 1:max_j){s_steps[j] <- s_lower + (j-1)*s_range/(max(1,max_j-1))}

rownames(sum_of_squares) <- m_steps

colnames(sum_of_squares) <- s_steps

for (i in 1:max_i){

for (j in 1:max_j){

sum_of_squares[i,j] <- 0

sum_of_squares[i,j] <- sum_of_squares[i,j] + sum((exp_data_2[2,]-100*pnorm(exp_data_2[1,],m_steps[i],s_steps[j]))^2)

if(max_dist<sum_of_squares[i,j]){

max_dist <- sum_of_squares[i,j]

max_dist_i <- i

max_dist_j <- j

}

}

}

min_dist <- max_dist

min_dist_i <- max_dist_i

min_dist_j <- max_dist_j

for (i in 1:max_i){

for (j in 1:max_j){

if(min_dist>sum_of_squares[i,j]){

min_dist <- sum_of_squares[i,j]

min_dist_i <- i

min_dist_j <- j

}

}

}

m_lower <- ifelse(min_dist_i==1,m_lower,m_steps[min_dist_i-1])

m_upper <- ifelse(min_dist_i==max_i,m_upper,m_steps[min_dist_i+1])

s_lower <- ifelse(min_dist_j==1,s_lower,s_steps[min_dist_j-1])

s_upper <- ifelse(min_dist_j==max_j,s_upper,s_steps[min_dist_j+1])

m_range <- m_upper-m_lower

s_range <- s_upper-s_lower

}

## the script outputs the mean and the standard deviation of the gaussian distribution

## best fitting the experimental distribution provided as input

print(c(m_lower,s_lower))
