# Supplementary material for: Contrasted Patterns of Crossover and Non-crossover at Arabidopsis thaliana Meiotic Recombination Hotspots
Source: PLoS Genet. 2013 Nov 14;9(11):e1003922. doi: 10.1371/journal.pgen.1003922 (PMC3828143; doi:10.1371/journal.pgen.1003922)
Supplement: Table S1 — Sequences of primers. (DOC) [file pgen.1003922.s006.doc]

**Table S1. Sequences of primers**

| Name | Sequence 5'->3' | Genomic coordinates |
| --- | --- | --- |
| 130x0CoL1 | CGCACACTCTTATCCAACA | 171696..171714 |
| 130x0LeL1 | CGCACATTCTTTTCCAACT | 171696..171714 |
| 130x7CoL4 | GCCTAATGTTCCCATGGATCTC | 173199..173220 |
| 130x7LeL5 | TGCCTAATGTTCCCATATCGAAG | 173198..173220 |
| 130x21CoL1 | CGACCAAGATTGGATAAAAAG | 176005..176025 |
| 130x21LeL5 | TCGACCAAGATTGGAAC | 176004..176033 |
| 130x21CoR5 | GGTACTGTTCCAATCTTTTTATC | 176039..176017 |
| 130x21LeR6 | GCAAGGTACTGTTCCAACCTCGG | 176043..176007 |
| 130x42LeL1 | GGTTACAGAGAATTGTAGAATGTTAC | 178103..178128 |
| 130x43CoL1 | GAAGAATGGATTATATAATACAGTACAGTATAT | 178138..178170 |
| 130x44CoL4 | AGTGGTCACTGTCGCTATAG | 178344..178363 |
| 130x44LeL4 | AAGTGGTCACTGTCGTTATAC | 178343..178363 |
| 130x44CoR1 | CAGCCCAATAGAATCCTATAG | 178378..178358 |
| 130x44LeR4 | CAGCCCAATAGAATCGTATAA | 178378..178358 |
| 130x47CoR2 | TGAGTCAATTAACGTGGTTTGCA | 179054..179032 |
| 130x47LeR4 | GTCGATGTCGTTGGTTTTCG | 179051..179032 |
| 130x52CoL1 | GAATcttgtctcacatgtctaga | 180401..180420 |
| 130x52CoR1 | TGTCAGAGATATTCTAGACATGTGA | 180432..180408 |
| 130x52LeR2 | TGGGATGAATTTTGTCAGAGCTATGA | 180444..180408 |
| 130x72CoR2 | GGGCCAATACATTGGAAACA | 185687..185668 |
| 130x72LeR2 | CAAGGGCCAAGACCTCAATC | 185690..185666 |
| 130x76CoR1 | tttcttcctatactcgtctcag | 185981..185960 |
| 130x78LeR3 | GGTTGGCCTCTTCTG | 186005..185991 |
| 14a8CoL3 | TATGCAACAGCAACACG | 16691260..16691275 |
| 14a5LeL3 | CCCCCCGCATGCTTTTACATTATA | 16691245..16691263 |
| 14a9Col2 | ACCGTTGCACTTTCCTT | 16692501..16692519 |
| 14a9LeL2 | CCGAAACCGTTGCACTATG | 16692500..16692530 |
| 14a23CoL1 | CCCCCGATTTATTCATACATATC | 16692993..16693014 |
| 14a23LeL1 | CCCCCGATTTATTCGTACATATT | 16692993..16693014 |
| 14a54CoR2 | GGAGAGCTAATGCAGGC | 16699799..16699783 |
| 14a54LeR2 | GCAGAGCCAATGCGGGT | 16699799..16699783 |
| 14a63CoR3 | CCTTTTGTTCGTACAGGTG | 16700136..16700120 |
| 14a63LeR3 | CCCCCATTTTGTTCGCAAAGGTA | 16700137..16700120 |
| 14aKH1L | ATCTTATAAACGTTATTGTCA | 16692865..16682885 |
| 14aKH2bisR | TTCGCCCGGCAAACTATTCC | 16695220..16695202 |
