# Supplementary material for: Contrasted Patterns of Crossover and Non-crossover at Arabidopsis thaliana Meiotic Recombination Hotspots
Source: PLoS Genet. 2013 Nov 14;9(11):e1003922. doi: 10.1371/journal.pgen.1003922 (PMC3828143; doi:10.1371/journal.pgen.1003922)
Supplement: Table S2 — Primers for DNA quantification and CO detection. (DOC) [file pgen.1003922.s007.doc]

**Table S2. Primers for DNA quantification and CO detection.**

| First PCR | Annealing temperature (°C) | Second PCR | Annealing temperature (°C) | Target:  Parental or CO |
| --- | --- | --- | --- | --- |
| 130x0CoL1-130x76CoR1 | 61 | 130x7CoL4-130x72CoR2 | 58 | parental |
| 130x0LeL1-130x78LeR3 | 58 | 130x7LeL5-130x72LeR2 | 58 | parental |
| 130x0CoL1-78LeR3 | 59 | 130x7CoL4-130x72LeR2 | 64 | CO |
| 130x0LeL1-130x76CoR1 | 59 | 130x7LeL5-130x72CoR2 | 64 | CO |
| 130x0CoL1-130x52LeR2 | 59 | 130x7CoL4-130x47LeR4 | 59 | CO |
| 130x0LeL1-130x52CoR1 | 59 | 130x7LeL5-130x47CoR2 | 59 | CO |
| 130x43CoL1-130x78LeR3 | 57 | 130x44CoL4-130x72LeR2 | 60 | CO |
| 130x42LeL1-130x78CoR2 | 58 | 130x44LeL4-130x72CoR2 | 58 | CO |
| 14a9Col2-14a63CoR3 | 58 | 14a23CoL1-14a54CoR2 | 58 | parental |
| 14a9LeL2-14a63LeR3 | 58 | 14a23LeL1-14a54LeR2 | 58 | parental |
| 14a8CoL3-14a63LeR3 | 61 | 14a9Col2-14a54LeR2 | 58 | CO |
| 14a5LeL3-14a63CoR3 | 61 | 14a9LeL2-14a54CoR2 | 58 | CO |
|  |  |  |  |  |
