# Supplementary material for: Contrasted Patterns of Crossover and Non-crossover at Arabidopsis thaliana Meiotic Recombination Hotspots
Source: PLoS Genet. 2013 Nov 14;9(11):e1003922. doi: 10.1371/journal.pgen.1003922 (PMC3828143; doi:10.1371/journal.pgen.1003922)
Supplement: Table S3 — Primers for NCO detection at 130×. (DOC) [file pgen.1003922.s008.doc]

**Table S3. Primers for NCO detection at 130x.**

| **Ler to Col to Ler** | | | |
| --- | --- | --- | --- |
| First PCR | Annealing temperature (°C) | Second PCR | Annealing temperature (°C) |
| 130x0LeL1-130x78LeR3 | 58 | 130x7LeL5-130x44CoR1 | 66 |
|  |  | 130x44CoL4-130x52LeR2 | 61 |
|  |  | 130x7LeL5-130x52CoR1 | 66 |
|  |  | 130x52CoL1-130x72LeR2 | 59 |
|  |  | 130x7LeL5-130x21CoR5 | 63 |
|  |  | 130x21CoL1-130x44LeR4 | 64 |

| **Col to Ler to Col** | | | |
| --- | --- | --- | --- |
| First PCR | Annealing temperature (°C) | Second PCR | Annealing temperature (°C) |
| 130x0CoL1-130x76CoR1 | 61 | 130x7CoL4-130x44LeR4 | 62 |
|  |  | 130x44LeL4-130x52CoR1 | 61 |
|  |  | 130x7CoL4-130x21LeR6 | 60 |
|  |  | 130x21LeL5-130x44CoR1 | 63 |
