# Supplementary material for: Contrasted Patterns of Crossover and Non-crossover at Arabidopsis thaliana Meiotic Recombination Hotspots
Source: PLoS Genet. 2013 Nov 14;9(11):e1003922. doi: 10.1371/journal.pgen.1003922 (PMC3828143; doi:10.1371/journal.pgen.1003922)
Supplement: Table S4 — Primers for NCO detection at 14a1 hotspot. (DOC) [file pgen.1003922.s009.doc]

**Table S4. Primers for NCO detection at 14a1 hotspot**

| SNP | Oligonucleotides | |
| --- | --- | --- |
|  | Name | Sequence |
| #35 | Y-1201-Col | GAAGGTCGGAGTCAACGGATTTTTCTTTTCGGCTTTTTCGG |
|  | Y-1201-Ler | GAAGGTGACCAAGTTCATGCTATTTCTTTTCGGCTTTTTCGA |
|  | Y-1021-Rev | GTGCCCTGCCATAATTCCACT |
| #37 | Y-1675-Col | GAAGGTGACCAAGTTCATGCTAGACTGATCGTTACCGATTCCG |
|  | Y-1675-Ler | GAAGGTCGGAGTCAACGGATTAGACTGATCGTTACCGATTCCA |
|  | Y-1675-Rev | GGTGGGTCCGAGTTTTCTTTGAT |
|  | M-768-Col | GAAGGTGACCAAGTTCATGCTTCTTAGTCAAACAATGGCTTAG |
| #33 | M-768-Ler | GAAGGTCGGAGTCAACGGATTCTTAGTCAAACAATGGCTTAT |
|  | M-768-Rev | ATGGCTGTTTTCGTTTCCTTAT |
